# Beta-galactosidase gene family genome-wide identification and expression analysis of members related to fruit softening in melon (*Cucumis melo* L.)

**DOI:** 10.1186/s12864-022-09006-5

**Published:** 2022-12-02

**Authors:** Haobin Pan, Yinhan Sun, Miaomiao Qiao, Hongyan Qi

**Affiliations:** 1 College of Horticulture, Shenyang Agricultural University, No.120 Dongling Road, Shenhe District, Shenyang, Liaoning 110866 People’s Republic of China; 2 Key Laboratory of Protected Horticulture of Education Ministry and Liaoning Province, Shenyang, Liaoning 110866 People’s Republic of China; 3 Northern National & Local Joint Engineering Research Center of Horticultural Facilities Design and Application Technology (Liaoning), Shenyang, Liaoning 110866 People’s Republic of China

**Keywords:** β-galactosidase gene, Bioinformatics, *Cucumis melo* L., Fruit softening, Genome-wide identification, Quantitative real-time PCR, Tissue-specific expression

## Abstract

**Background:**

Texture quality is impotent for melon (*Cucumis melo* L.) fruit. β-galactosidase (β-Gal, EC 3.2.1.23) is an important cell wall glycosyl hydrolase involved in fruit softening, However, the β-Gal gene (*BGALs*) family hasn’t been identified genome-wide in melon. Thus, it’s necessary to conduct an in-depth bioinformatic analysis on melon *BGALs* family and to seek out the key members who participated in melon fruit softening.

**Results:**

A total of 21 *BGALs* members designated as *CmBGAL1-CmBGAL21* were identified genome-wide in melon, clustered into A-G seven clades. Among them, three duplications *CmBGAL1*:*CmBGAL3*, *CmBGAL19*:*CmBGAL21*, and *CmBGAL20*:*CmBGAL21* happened*.* For conserved domains, besides the Glyco_hydro_35 domain (PF01301), all the members also contained the GHD domain (PF17834) except for CmBGAL12, and the Gal_Lectin (PF02140) domain existed in most CmBGALs at the C-termini. Motifs, protein secondary and tertiary structure analysis showed that the CmBGAL12 is a unique member. Moreover, protein-protein association network analysis showed that the CmBGAL12 is the only node protein. Furthermore, spatiotemporal expression pattern analysis by quantitative real-time PCR (qRT-PCR) suggested that most of *CmBGAL*s expressed in tissues with vigorous cell wall remodeling/disassembly. In addition, *cis*-acting regulatory elements analysis in promoters inferred that *CmBGALs* might participate in diverse responsiveness to phytohormone, biotic and abiotic signaling.

**Conclusions:**

A novel clade of *CmBGAL* members (Clade F) related to melon fruit softening was discovered, since their expression showed a specific surge in the mature fruit of ‘HPM’ with mealy texture (softening sharply), but not in ‘HDB’ with crisp texture (softening bluntly). The homologous *CmBGAL7–11* in Clade F exhibited identical spatiotemporal expression patterns may multiple genes leading to melon fruit softening.

**Supplementary Information:**

The online version contains supplementary material available at 10.1186/s12864-022-09006-5.

## Background

Melon (*Cucumis melo* L.) is a kind of typical climacteric fleshy fruit, and texture is important for evaluating the commercial quality for it. Moreover, the softening during fruit ripening and postharvest storage which decides the transportability and shelf-life. So, it’s meaningful to illuminate the mechanism of fruit softening. In the latest decades, it has been elucidated that the cell wall polysaccharides modification and disassembly is the initial reason for fruit softening [1], and varieties of hydrolytic enzymes, like polygalacturonase (PG, EC 3.2.1.15), pectin methylesterase (PME, EC 3.1.1.11), β-Gal, etc. participated in this process [2]. However, it is still unclear which are the key enzymes involved in melon fruit softening.

β-Gal is a kind of glycosyl hydrolase, and its role in fruit softening has been reported in apple [3], tomato [4, 5], muskmelon [6], avocado [7], kiwifruit [8], Japanese pear [9] and papaya [10]. β-Gal could remove the β-D-galactosyl residues from the non-reducing terminal of pectin and hemicellulose polymers like rhamnogalacturonan-I (RG-I) galactan side chains, xyloglucan, galactolipids and glycoprotein by cutting β-(1, 2)-, β-(1, 3)-, β-(1, 4)- or β-(1, 6)-glycosidic bonds to increase the porosity of cell wall and enhancing the access of other cell wall-degrading enzymes to accelerate fruit softening [2, 11, 12]. Meantime, β-Gal also widely participated in the biological processes including seed germination [13, 14], organ elongation [15, 16] etc. related to cell wall remodeling.

In this study, the β-Gal activity and *BGALs* expression in fruit were compared between two melon cultivars ‘HDB’ (Crisp) and ‘HPM’ (Mealy) which exhibited blunt and sharp softening respectively during development. Since previous study in apple fruit softening showed that the β-Gal activity in ‘Fuji’ (Soft & Crisp) was continuously higher than that in ‘Qinguan’ (Firm & Tough), especially at the mature stage. Meantime, the expression level of *Mdβ-Gal1*, *Mdβ-Gal2* and *Mdβ-Gal5* increased dramatically and significantly higher in ‘Fuji’ than that in ‘Qinguan’ at the later ripening [17]. In peach, it was also observed that the *PpBGAL2* and *PpBGAL16* exhibited significantly different expression during fruit postharvest softening between four cultivars with different softening characteristics [18]. In addition, the *TBG4* in tomato [19], and the *FaβGal4* in strawberry [20] also have been verified contributing to fruit softening by transgene. However, some studies showed that not all the isoforms of β-Gal had exo-galactanase activity, and the different isoforms of β-Gal are specific to different cell wall substrates [5, 6]. Therefore, it is necessary to identify the BGALs family members and seek out the key members relating to fruit softening.

The BGALs belong to the glycosyl hydrolase 35 (GH35) family, possessing an exclusive consensus sequence of active site, G-G-P-[LIVM](2)-x(2)-Q-x-E-N-E-[FY] [21]. Up to now, the plant *BGALs* family have been identified in *Pyrus pyrifolia* (8) [22], *Arabidopsis thaliana* (17) [23], *Persea americana* Mill. (4) [24], *Oryza sativa* L. (15) [25], *Brassica campestris* ssp. *chinensis* (27) [26], *Linum usitatissimum* (43) [27], *Fragaria ananassa* (4) [20, 28], *Solanum lycopersicum* (17) [29, 30], *Prunus persica* (L.) Batsch (17) [18], *Malus domestica* L. (13) [17] and *Lpomoea batatas* (L.) Lam (17) [31], they are all multigene family. However, which of these members plays a key role in fruit softening is still not totally clear. Hence, we decided to identify the *BGALs* family in *Cucumis melo* L., and to give an in-depth bioinformatic analysis and qRT-PCR expression analysis on it, aiming to explore the key BGAL members involved in melon fruit softening.

## Results

### Identification of melon *BGAL* genes and phylogenetic analysis

A total of 21 *BGAL* genes were identified from the melon genome. These genes were designated as *CmBGAL1-CmBGAL21* according to the homology with reported genes*.* The gene information of *CmBGALs* were analyzed (Table 1). In general, the length of CDS ranged from 2094 (*CmBGAL13*) to 2823 (*CmBGAL11*) bp, and the length of deduced protein sequences ranged from 697 to 940 aa with Mw of 78,652.05 to 105,903.53 kDa. Moreover, the pI varied from 5.2 (CmBGAL6) to 9.19 (CmBGAL5), and the GRAVY varied from − 0.501 (CmBGAL5) to − 0.087 (CmBGAL12), all showed hydrophilic property. Additionally, the results of protein subcellular location prediction demonstrated that the majority of CmBGALs are located in extracellular space.

**Table 1:** *BGAL* genes in *Cucumis melo* L. and their annotated information

Clade^a^	Name^b^	Accession^c^	Chromosome location^d^	CDS (bp)^e^	Protein (aa)^f^	Mw (kDa)^g^	Theoretical pI^h^	GRAVY^i^	Subcellular location
A	*CmBGAL1*	MELO3C013055.2	chr04: 16717893 ~ 16,724,281 (+)	2499	832	92,701.76	7.97	−0.245	plasma membrane
	*CmBGAL2*	MELO3C015471.2	chr02: 2015336 ~ 2,018,327 (+)	2160	719	80,740.12	8.62	− 0.284	extracellular space
	*CmBGAL3*	MELO3C015469.2	chr02: 1973422 ~ 1,979,527 (−)	2115	704	78,350.45	6.94	−0.202	extracellular space
	*CmBGAL4*	MELO3C015470.2	chr02: 2002073 ~ 2,007,432 (+)	2172	723	80,714.24	8.24	−0.224	plasma membrane
B	*CmBGAL18*	MELO3C023335.2	chr11: 1715509 ~ 1,722,003 (+)	2538	845	95,018.95	8.08	−0.353	extracellular space
	*CmBGAL19*	MELO3C003792.2	chr04: 4395761 ~ 4,401,392 (−)	2535	844	93,015.73	8.33	−0.216	extracellular space
	*CmBGAL20*	MELO3C016409.2	chr07: 24227139 ~ 24,234,240 (+)	2541	846	94,412.33	7.78	−0.235	extracellular space
	*CmBGAL21*	MELO3C007872.2	chr08: 5904316 ~ 5,910,615 (+)	2565	854	94,637.49	7.24	−0.237	plasma membrane
C	*CmBGAL16*	MELO3C005054.2	chr12: 3822196 ~ 3,829,733 (−)	2097	698	76,687.45	6.37	−0.244	plasma membrane
	*CmBGAL17*	MELO3C006301.2	chr06: 2352074 ~ 2,364,404 (+)	2523	840	94,121.93	6.51	−0.261	extracellular space
D	*CmBGAL13*	MELO3C023188.2	chr11: 5193 ~ 15,903 (+)	2094	697	78,652.05	7.00	−0.240	extracellular space
E	*CmBGAL14*	MELO3C010636.2	chr03: 8459760 ~ 8,467,653 (−)	2622	873	97,630.79	6.57	−0.332	extracellular space
	*CmBGAL15*	MELO3C013360.2	chr01: 16900517 ~ 16,905,789 (+)	2259	752	84,409.23	8.05	−0.359	extracellular space
F	*CmBGAL5*	MELO3C006540.2	chr06: 4015808 ~ 4,021,686 (+)	2562	853	96,873.30	9.19	−0.501	extracellular space
	*CmBGAL6*	MELO3C015540.2	chr02: 2657588 ~ 2,661,148 (+)	2496	831	93,519.70	5.20	−0.308	plasma membrane
	*CmBGAL7*	MELO3C012947.2	chr04: 14973846 ~ 14,976,326 (+)	2481	826	92,921.02	6.18	−0.339	extracellular space
	*CmBGAL8*	MELO3C026513.2	chr03: 24492677 ~ 24,495,192 (+)	2475	824	92,584.84	8.75	−0.396	extracellular space
	*CmBGAL9*	MELO3C033812.2	chr09: 21595815 ~ 21,598,283 (−)	2469	822	91,930.90	7.70	−0.339	extracellular space
	*CmBGAL10*	MELO3C009997.2	chr02: 11301269 ~ 11,303,743 (+)	2475	824	92,304.28	6.85	−0.339	extracellular space
	*CmBGAL11*	MELO3C015321.2	chr02: 743784 ~ 747,625 (+)	2823	940	105,903.53	6.74	−0.416	nucleus
G	*CmBGAL12*	MELO3C025840.2	chr04: 17683384 ~ 17,693,548 (+)	2205	734	83,458.13	7.69	−0.087	endomembrane system

^a^ Clade distribution according to phylogenetic clustering

^b^ Names given by nomenclature system to *BGAL* genes of *Cucumis melo* L

^c^ Gene accession of *CmBGALs* in CuGenDB

^d^ Gene chromosome location and direction, “+” means 5′-3′, “-” means 3′-5′

^e^ Length of coding sequence

^f^ Length of protein sequence

^g^ Molecular weight

^h^ Theoretical isoelectric point

^i^ Grand average of hydropathicity index

**Fig. 1:**
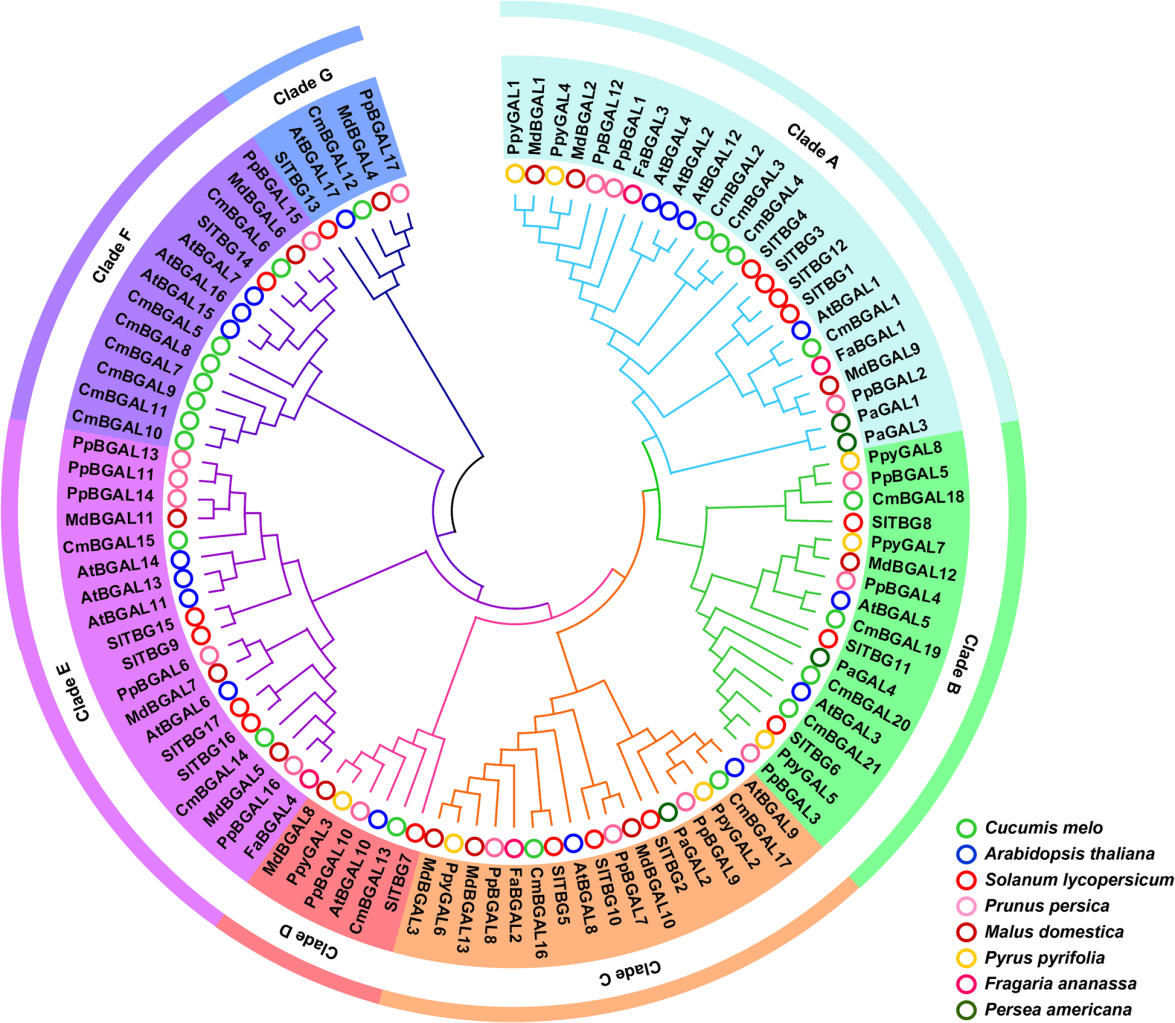
Phylogenetic tree of BGALs among *Cucumis melo*, *Arabidopsis thaliana*, *Solanum lycopersicum*, *Prunus persica*, *Malus domestica*, *Pyrus pyrifolia*, *Fragaria ananassa* and *Persea americana*. To distinguish the Latin name of *Prunus persica* from *Pyrus pyrifolia*, we abbreviated them as ‘Pp’ and ‘Ppy’, respectively. The gene accession numbers of all the BGAL genes are shown in Table S2

Furthermore, a phylogenetic tree of the BGALs of *Cucumis melo* (21)*,* *Arabidopsis thaliana* (17), *Solanum lycopersicum* (17)*,* and other fleshy fruit species *Prunus persica* (17)*, Malus domestica* (13)*, Pyrus pyrifolia* (8), *Fragaria ananassa* (4) and *Persea americana* (4) was constructed to illustrate the evolutionary relationships among them (Fig. 1). Finally, all these BGALs were clustered into seven clades (A-G), Clade F contains the most members of CmBGAL (seven: CmBGAL5–11), Clades A and B each has four members (CmBGAL1–4; CmBGAL18–21), Clades D and E each has two members (CmBGAL16, 17; CmBGAL14, 15), and Clades C and G each has one member (CmBGAL13; CmBGAL12).

### Gene structure analysis of *CmBGAL*s

**Fig. 2:**
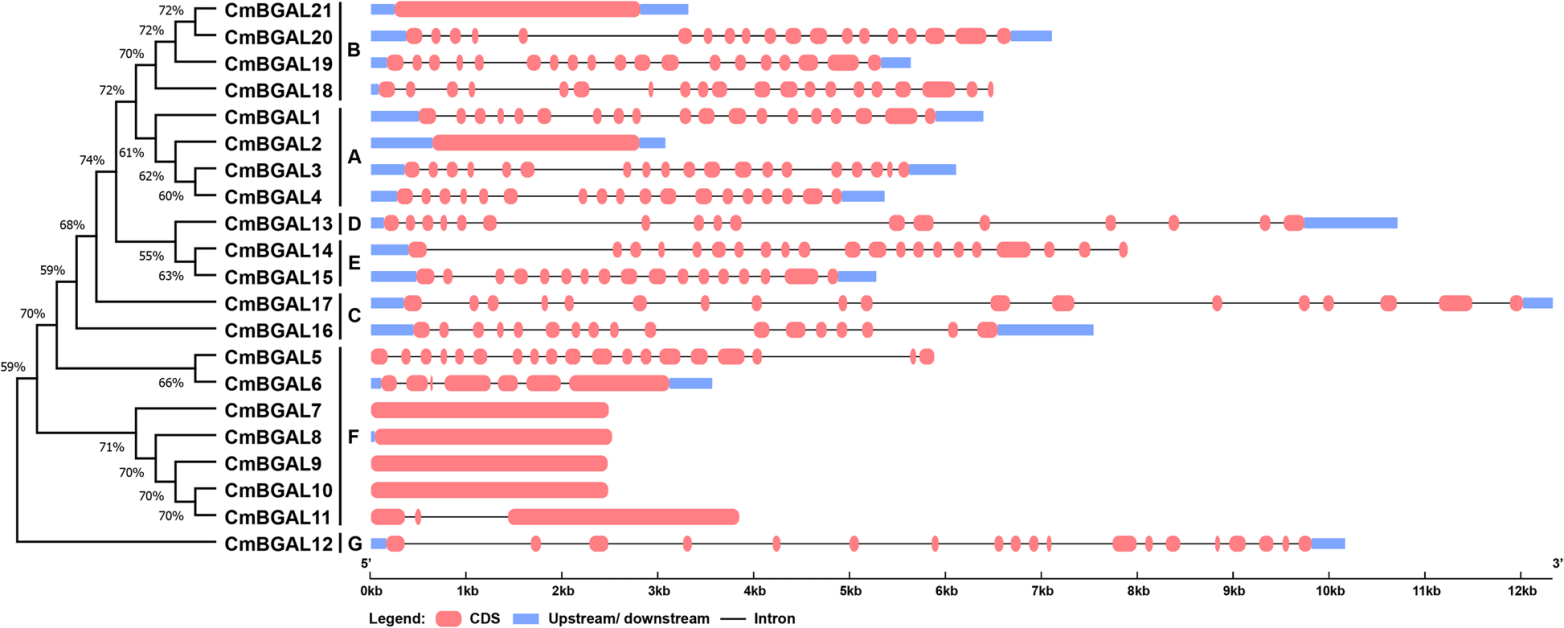
Phylogenetic and gene structure analysis of *CmBGAL* members

Gene structure combined phylogenetic tree among *CmBGALs* family members were visualized based on gene CDS and corresponding sequences with intron. The results showed that the structure of *CmBGAL*s exhibited high divergence. While it is worth noting that the members in Clade F with fewer introns, especially *CmBGAL7–11* (Fig. 2).

### Chromosomal location and gene duplication analysis of *CmBGAL*s

**Fig. 3:**
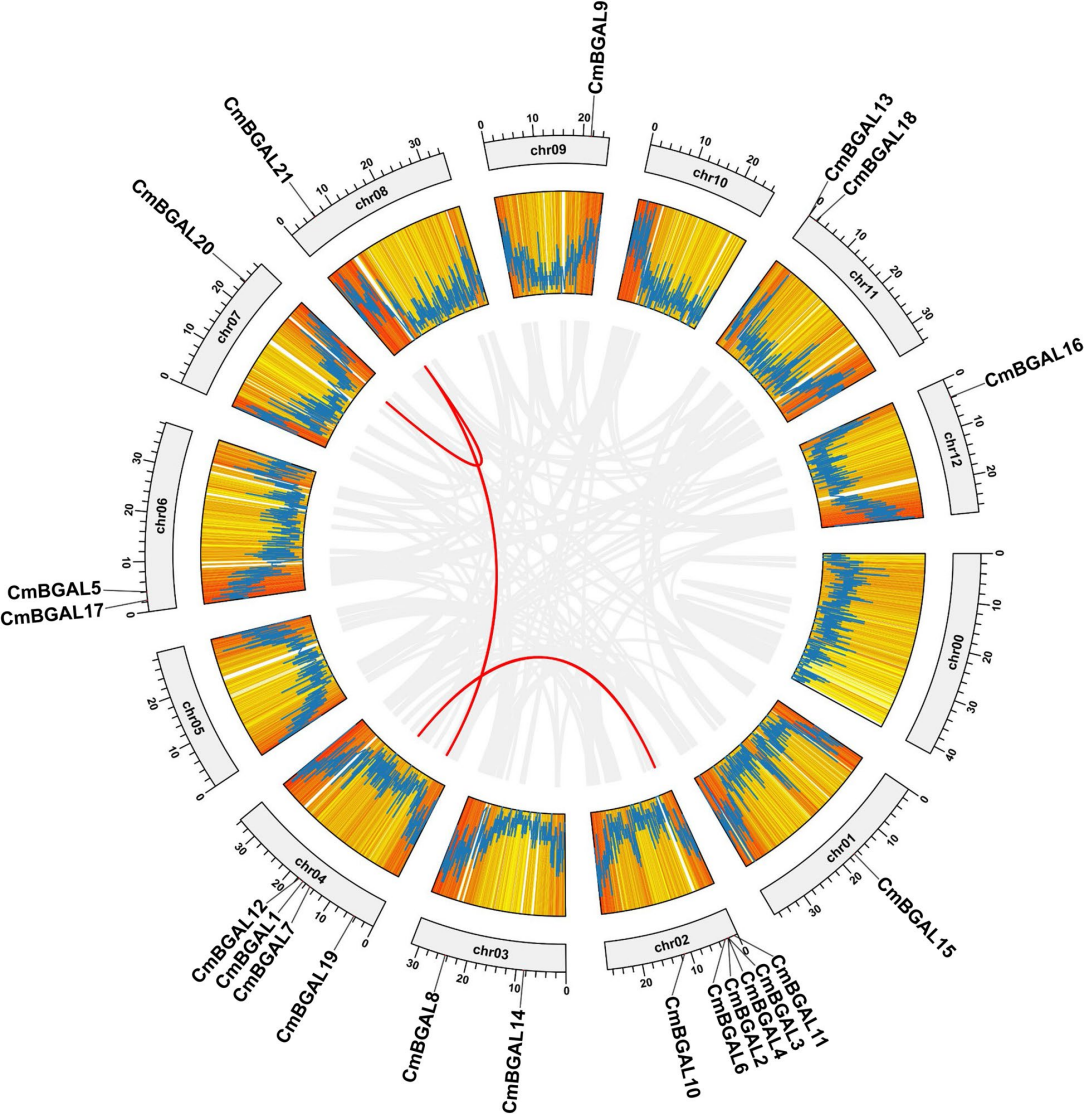
Chromosomal location and gene duplication analysis of *CmBGAL* members

The chromosomal location displayed that 21 *CmBGALs* distributes unevenly on 10 of 12 different chromosomes in melon. Chr02 owns the most *CmBGAL* members, with six, followed by Chr04, with four. Chr03, Chr06 and Chr11 each owns two members, and Chr01, Chr07, Chr08, Chr09 and Chr12 each owns one. No location site was found on Chr05 and Chr10 (Fig. 3).

Meanwhile, three segmental duplication gene pairs were found among 21 *CmBGAL* members by syntenic analysis, they were *CmBGAL1*:*CmBGAL3*, *CmBGAL19*:*CmBGAL21* and *CmBGAL20*:*CmBGAL21,* suggesting that there exist specific evolution and biological function relationships between them.

### Conserved domains and motifs analysis of CmBGALs

**Fig. 4:**
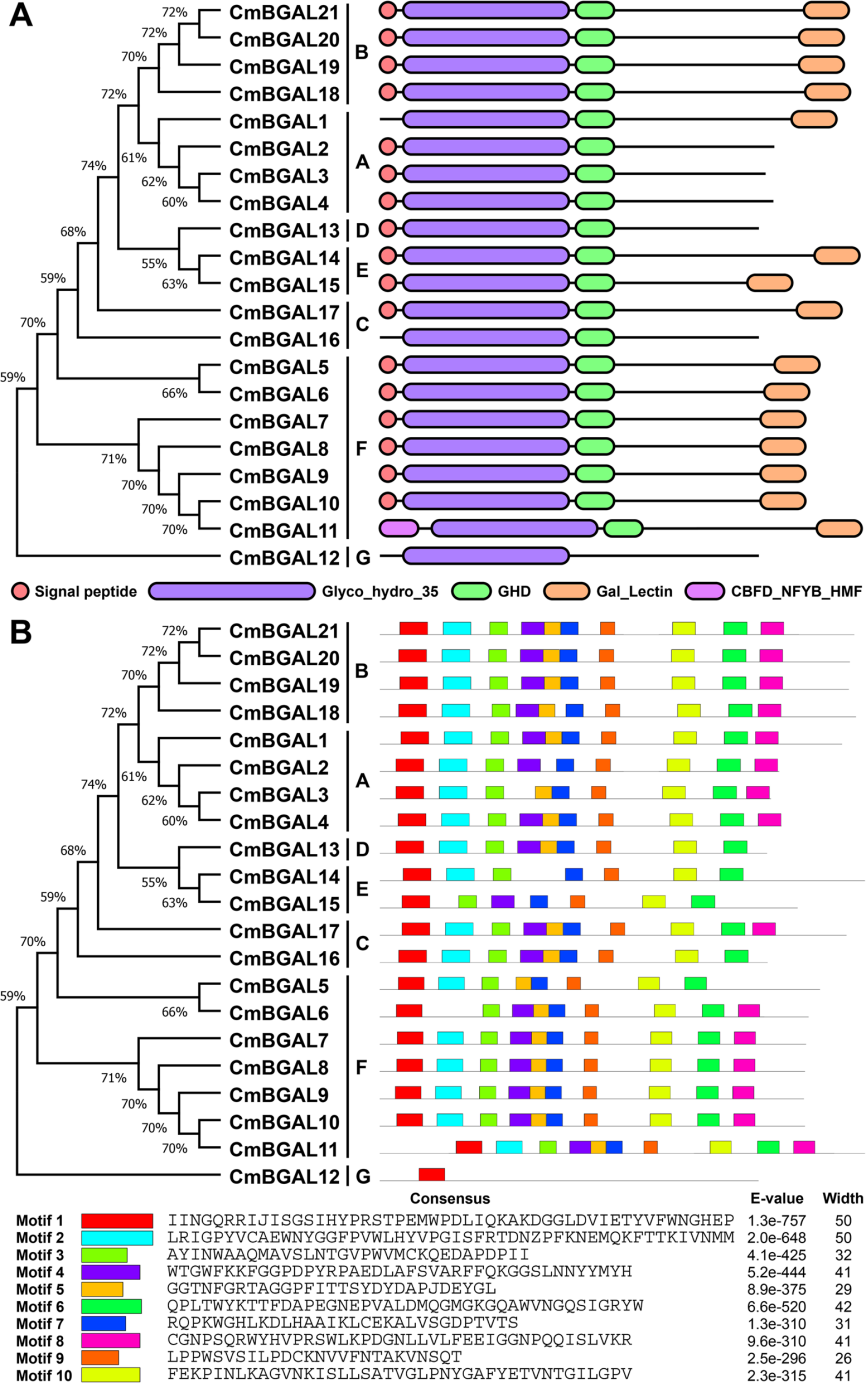
Phylogenetic with conserved domains and motifs distribution of CmBGALs. **A** The conserved domains distribution in CmBGALs; **B** The motifs distribution in CmBGALs

The conserved domains and signal peptide in 21 CmBGALs were analyzed by the NCBI CDD website (Fig. 4A) which verified that all the 21 CmBGALs contain the Glyco_hydro_35 domain (PF01301) with the characteristic active site consensus sequence G-G-P-[LIVM](2)-x(2)-Q-x-E-N-E-[FY] for BGAL. In addition, except for CmBGAL12, all the CmBGAL members containe the GHD domain (PF17834). Besides that, the Gal_Lectin domain (PF02140) distributes on the C-termini of CmBGAL members except for CmBGAL2, CmBGAL3, CmBGAL4, CmBGAL12, CmBGAL13 and CmBGAL16. Interestingly, a special CBFD_NFYB_HMF (PF00808) domain N-terminus was only found in CmBGAL11. The multiple sequence alignment of amino acid sequences exhibiting the position and consensus of the above domains in CmBGALs (Fig. S1). The conserved domains information in CmBGALs was showed in Table S1. Apart from that, In the 21 CmBGALs, 17 are predicted to have an N-terminal signal peptide that targets the protein to the plasma membrane or endomembrane system.

In addition, we also analyzed the composition of motifs for CmBGALs (Fig. 4B). The ten most conserved motifs were identified. The results showed that most CmBGAL members containe Motif 1–10, but also existing absence. CmBGAL6 and CmBGAL15 lack of Motif 2, CmBGAL3, CmBGAL5 and CmBGAL14 lack of Motif 4, CmBGAL2, CmBGAL14 and CmBGAL15 lack of Motif 5, and CmBGAL5, CmBGAL13, CmBGAL14, CmBGAL15 and CmBGAL17 lack of Motif 8. Distinctively, the CmBGAL12 only has Motif 1.

### Secondary and tertiary structure prediction of CmBGALs

The prediction of secondary structure for CmBGALs reveals that the random coil accounts for the highest percentage among the secondary structure, ranging from 42.42% (CmBGAL15) to 46.73% (CmBGAL12). The extended strand ranging from 20.98% (CmBGAL12) to 27.84% (CmBGAL3), followed by α-helix ranging from 18.78% (CmBGAL2) to 25.07% (CmBGAL12). β-turn accounted for the lowest, ranging from 6.76% (CmBGAL14) to 9.09% (CmBGAL3) (Table 2).

**Table 2:** Protein secondary structure of CmBGALs

Clade	Protein	Protein secondary structure			
		α-Helix	β-Turn	Random coil	Extended strand
A	CmBGAL1	20.55%	7.69%	46.39%	25.36%
	CmBGAL2	18.78%	8.90%	45.34%	26.98%
	CmBGAL3	19.46%	9.09%	43.61%	27.84%
	CmBGAL4	19.92%	8.71%	45.37%	26.00%
B	CmBGAL18	19.17%	7.57%	46.75%	26.51%
	CmBGAL19	20.50%	8.53%	45.85%	25.12%
	CmBGAL20	19.39%	8.16%	46.45%	26.00%
	CmBGAL21	20.49%	7.73%	46.14%	25.64%
C	CmBGAL16	20.06%	8.45%	44.99%	26.50%
	CmBGAL17	21.19%	7.62%	46.19%	25.00%
D	CmBGAL13	20.80%	8.18%	44.91%	26.11%
E	CmBGAL14	23.14%	6.76%	43.30%	26.80%
	CmBGAL15	22.34%	7.85%	42.42%	27.39%
F	CmBGAL5	22.74%	8.91%	42.67%	25.67%
	CmBGAL6	19.86%	8.30%	45.49%	26.35%
	CmBGAL7	20.82%	8.23%	44.92%	26.03%
	CmBGAL8	20.27%	8.62%	45.27%	25.85%
	CmBGAL9	20.56%	8.03%	45.38%	26.03%
	CmBGAL10	21.48%	8.01%	44.42%	26.09%
	CmBGAL11	23.30%	7.77%	44.04%	24.89%
G	CmBGAL12	25.07%	7.22%	46.73%	20.98%

**Fig. 5:**
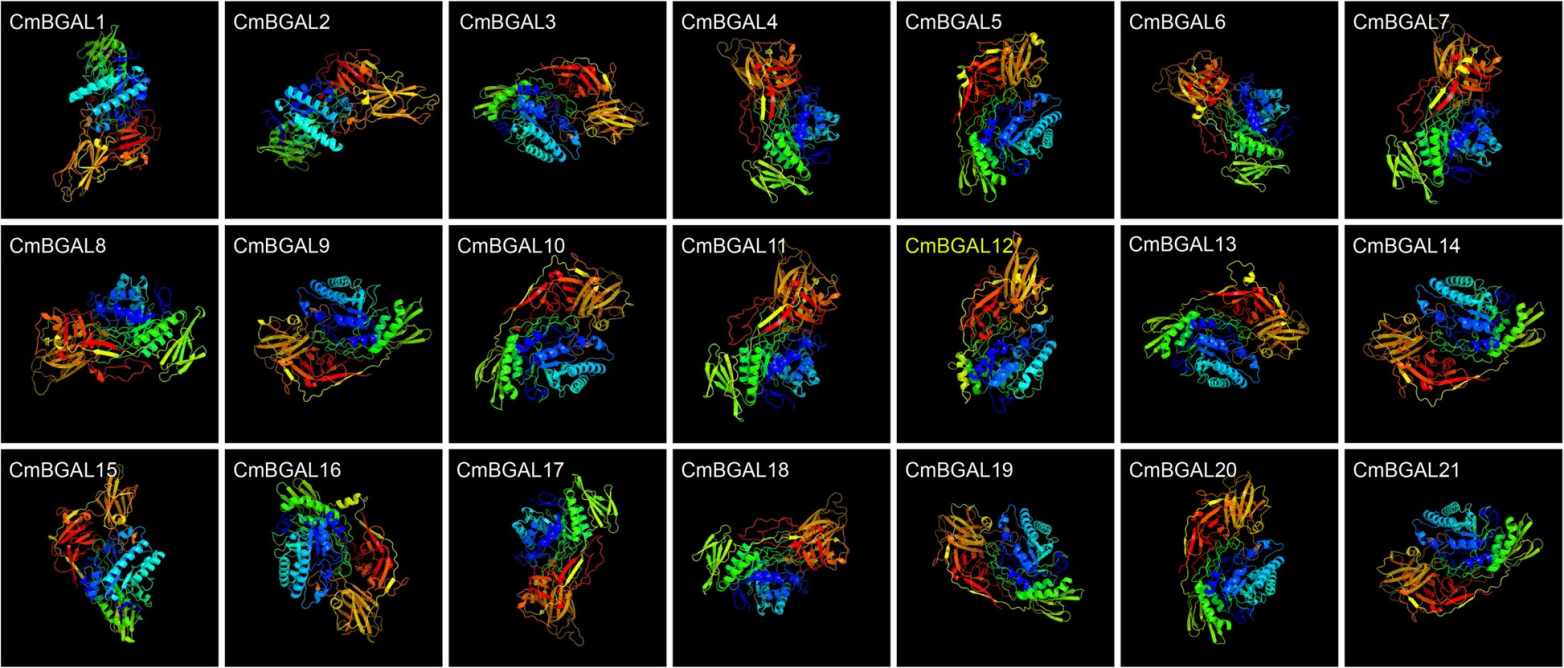
Tertiary structure of CmBGALs predicted by homologous modeling. The 3D model of CmBGALs named in white color are based on the ‘c3w5gB’ template (https://www.ebi.ac.uk/pdbe/entry/pdb/3w5g), in yellow color is based on the ‘c6eonA’ template (https://www.ebi.ac.uk/pdbe/entry/pdb/6eon)

To further compare the protein tertiary structures among the 21 CmBGAL members, the protein 3D models were constructed by homologous modeling (Fig. 5). The 3D model of all CmBGAL member proteins were based on the ‘c3w5gB’ template, except for CmBGAL12 which based on the ‘c6eonA’ template, indicating that the protein function of CmBGAL12 differs from other members.

### Protein-protein association network analysis of CmBGALs

**Fig. 6:**
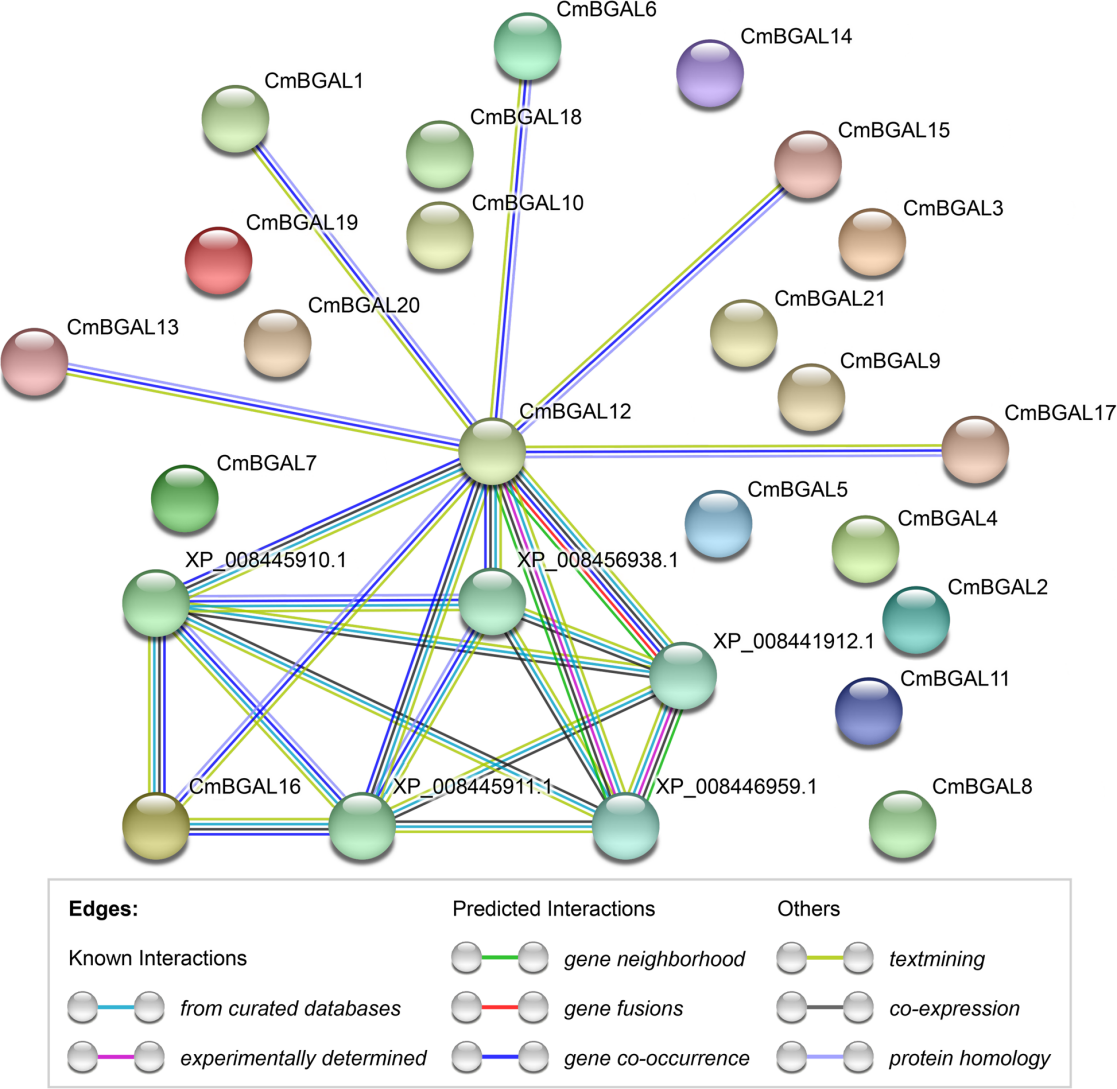
Protein-protein association network analysis of CmBGALs. XP_008445910.1, XP_008445911.1, XP_008441912.1, XP_008446959.1 and XP_008456938.1 are the protein accession numbers in GenBank (https://www.ncbi.nlm.nih.gov/genbank/)

The STRING protein association network among CmBGAL members showed that the CmBGAL12 is associated with CmBGAL1, CmBGAL6, CmBGAL13, CmBGAL15, CmBGAL16 and CmBGAL17 in gene co-occurrence, textmining and protein homology. The other CmBGAL members are isolated from each other. In addition, we discovered two alpha-galactosidase proteins (XP_008445910.1 and XP_008445911.1) as the commonly association nodes between CmBGAL12 and CmBGAL16 in curated databases, gene co-occurrence, textmining and co-expression. Furthermore, CmBGAL12 is also associated with a beta-hexosaminidase protein (XP_008441912.1), a mistakenly identified beta-galactosidase protein which belongs to the glycosyl hydrolase 2 family (XP_008446959.1) and another alpha-galactosidase protein (XP_008456938.1) (Fig. 6).

### Expression pattern analysis of *CmBGAL*s in various tissues

**Fig. 7:**
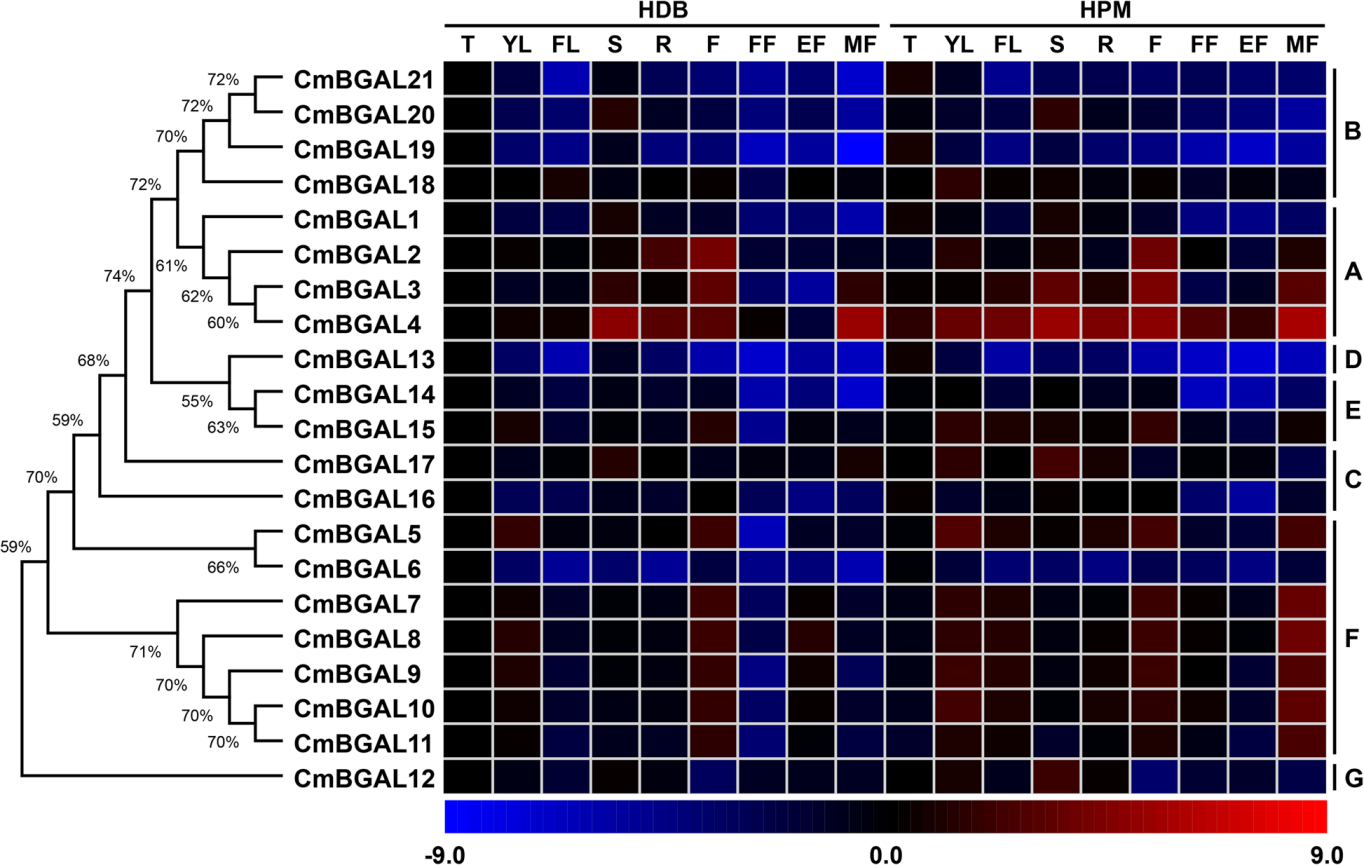
Heatmap of the spatiotemporal expression pattern of *CmBGAL* members in various tissues of ‘HDB’ and ‘HPM’, respectively. The values of relative expression were log_2_-transformed. Blue represents a low expression level, black represents a medium, and red represents a high level. T: tendrils; YL: young leaves; FL: functional leaves; S: stems; R: roots; F: flowers; FF: fruitlet fruits; EF: expanding fruits; MF: mature fruits

To assess the potential functions of *CmBGALs*, the spatiotemporal expression pattern of 21 *CmBGAL* members in various tissues including tendrils, young leaves, functional leaves, stems, roots, flowers and fruits at fruitlet, expanding and mature stage were compared between two cultivars of melon ‘HDB’ and ‘HPM’ (Fig. 7). The result suggested that most of the *CmBGALs* showed tissue-specific expression. In Clade A, *CmBGAL1* relatively higher expressed in tendril and stem, *CmBGAL2* and *CmBGAL3* showed specific expression in flower. In Clade B, *CmBGAL19* and *CmBGAL21* showed tendril-specific expression, *CmBGAL20* showed stem-specific expression. The *CmBGAL13* in Clade D showed tendril-specific expression. The *CmBGAL14* in Clade E showed extremely low expression level in fruit. Intriguingly, the expression of *CmBGAL7–11* in Clade F showed almost an identical spatiotemporal expression pattern, all of them specific expressed in the mature fruit of ‘HPM’, and also showed a relative higher expression level in flower, while the *CmBGAL5* and *CmBGAL6* also in Clade F showed different spatiotemporal expression patterns with them. Overall, the above results illustrated that the *CmBGALs* exert their functions in various tissues as well as diverse physiological processes in plant growth and development. The relative expression level data of 21 *CmBGAL* members is showed in Additional file 9.

### Expression analysis of putative fruit softening-related *CmBGAL* members in Clade A and F

**Fig. 8:**
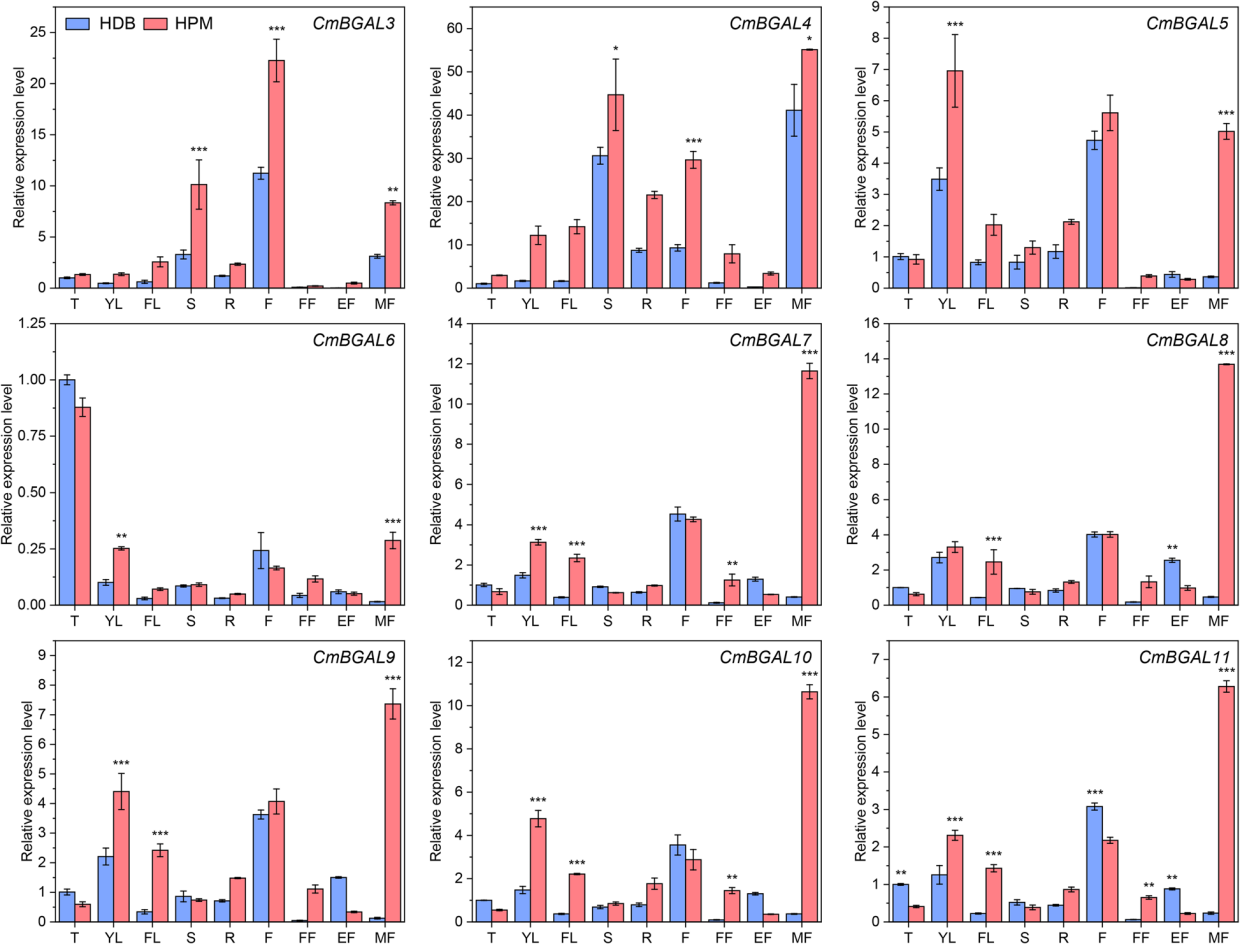
Relative expression level of putative fruit softening-related *CmBGAL* members in ‘HDB’ and ‘HPM’. T: tendrils; YL: young leaves; FL: functional leaves; S: stems; R: roots; F: flowers; FF: fruitlet fruits; EF: expanding fruits; MF: mature fruits. The vertical bars indicate the standard error of the means of triplicates. Significant differences between the means were compared by Tukey test with * *P* < 0.05, ** *P* < 0.01 and *** *P* < 0.001

To confirm the potential role of *CmBGAL* members in melon fruit softening, the genes expression, hardness and β-Gal activity were compared between the two texture types of melon ‘HDB’ and ‘HPM’. The hardness of ‘HPM’ fruit declined sharply from the S3 to S4, while that of ‘HDB’ fruit declined bluntly and kept significantly higher (*P* < 0.001) than that of ‘HPM’ fruit especially at the mature stage (Fig. S2). Meanwhile, according to the paired comparation analysis, nine *CmBGAL* members exhibited a significant difference in expression at the mature fruit between ‘HDB’ and ‘HPM’ were screened out, they are *CmBGAL3* (*P* < 0.01) and *CmBGAL4* (*P* < 0.05) in Clade A, and *CmBGAL5–11* (*P* < 0.001) in Clade F (Fig. 8). No significant difference was observed in any other members between the two cultivars.

The expression of the nine *CmBGAL* members in fruit can be divided into two patterns: 1) Rose at the mature stage in both ‘HDB’ and ‘HPM’ fruit (*CmBGAL3* and *CmBGAL4*); 2) Only surged in the mature fruit of ‘HPM’ (*CmBGAL5–11*). Therefore, we considered *CmBGAL3* and *CmBGAL4* as mature-respond genes (Compared to *CmBGAL3,* the *CmBGAL4* exhibited a more specific expression in mature fruit); While *CmBGAL5–11* as the genes contributing to softening behaviour difference between ‘HDB’ and ‘HPM’ fruits, especially the *CmBGAL7–11* with identical spatiotemporal expression patterns showed a predominant surge in the mature fruit of ‘HPM’. In addition, the activity of β-Gal in ‘HDB’ and ‘HPM’ fruit was measured, a significant increase in ‘HPM’ fruit at the mature stage was observed, but not in ‘HDB’ (Fig. S3). Furthermore, the correlation analysis between the expression level of *CmBGAL3–11* with hardness and β-Gal activity of fruit was conducted (Table 3). The correlation coefficients between *CmBGAL3–11* expression and hardness all exceed − 0.8, and their expression all showed different extent positive correlations with β-Gal activity, especially *CmBGAL5–11*.

**Table 3:** Pearson correlation coefficients between the relative expression level of *CmBGAL3–11* with hardness and β-Gal activity in fruit

	*CmBGAL3*	*CmBGAL4*	*CmBGAL5*	*CmBGAL6*	*CmBGAL7*	*CmBGAL8*	*CmBGAL9*	*CmBGAL10*	*CmBGAL11*
Hardness	−0.991**	−0.941	− 0.913	−0.841	− 0.895	− 0.869	−0.862	− 0.895	−0.892
β-Gal activity	0.217	0.065	0.388	0.607	0.411	0.373	0.399	0.420	0.388

Note: * and ** on the coefficients mean significance at the *P* < 0.05 and *P* < 0.01 level, respectively

### *Cis*-acting regulatory elements analysis in *CmBGAL* promoters

To further understand the *cis*-acting regulation of *CmBGALs*, the *cis*-acting regulatory elements in the promoters of each *CmBGAL* were analyzed except for *CmBGAL9* as the promoter sequence of it missed in both CuGenDB and GenBank (Table 4). The *cis*-acting regulatory elements in *CmBGALs* promoters were classed into four types: phytohormone responsive elements, stress responsive elements, light responsive elements, and other elements. Regarding phytohormone responsiveness, most of the *CmBGAL* promoters contain ethylene-responsive element (ERE) except for *CmBGAL8, CmBGAL13, CmBGAL17* and *CmBGAL21*. The promoters of *CmBGAL1–4, CmBGAL6, CmBGAL8, CmBGAL12, CmBGAL16* and *CmBGAL21* contain the TCA-element and *CmBGAL6* also contain SARE which are involved in salicylic acid responsiveness. CGTCA-motif or TGACG-motif which involved in the methyl jasmonate responsiveness were found in *CmBGAL1, CmBGAL2, CmBGAL4, CmBGAL6–8, CmBGAL12, CmBGAL13, CmBGAL17* and *CmBGAL19–21*. The gibberellin-responsive elements P-box, GARE-motif or TATC-box were found in *CmBGAL1–5, CmBGAL8, CmBGAL13, CmBGAL14, CmBGAL16, CmBGAL17, CmBGAL19* and *CmBGAL21.* The auxin-responsive elements TGA-element, TGA-box or AuxRR-core were found in *CmBGAL1, CmBGAL6–8, CmBGAL12, CmBGAL15, CmBGAL20* and *CmBGAL21*. The abscisic acid-responsive element ABRE were found in *CmBGAL3, CmBGAL5, CmBGAL10, CmBGAL11, CmBGAL12–17* and *CmBGAL19–21*. For stress responsiveness, all promoters of *CmBGAL* members contain ARE which is essential for the anaerobic induction except for *CmBGAL11* and *CmBGAL17.* The MYB binding site (MBS) involved in drought-inducibility was found in *CmBGAL2, CmBGAL4, CmBGAL5, CmBGAL13, CmBGAL15* and *CmBGAL19*. The LTR element involved in low-temperature responsiveness was found in *CmBGAL3, CmBGAL6, CmBGAL14, CmBGAL16, CmBGAL17* and *CmBGAL19*. The WUN-motif responds to wound was found in *CmBGAL1, CmBGAL7, CmBGAL12, CmBGAL14–16* and *CmBGAL18–21*. The TC-rich repeats involved in defense and stress responsiveness was found in *CmBGAL*2–4, *CmBGAL6, CmBGAL*13, *CmBGAL*16 and *CmBGAL*21. Plenty of light-responsive elements were found in the *CmBGALs* promoters, and the most frequently occurred were Box 4, G-box and GT1-motif. In addition, elements involved in meristem (CAT-box) and endosperm (GCN4_motif) expression, and palisade mesophyll cells differentiation (HD-Zip 1) were also found. These results inferring that *CmBGALs* participated in diverse responsiveness to hormone, biotic and abiotic signaling.

**Table 4:** *Cis*-acting regulatory elements in *CmBGAL* promoters

Clade	Gene	Phytohormone responsive elements	Stress responsive elements	Light responsive elements	Other elements
A	*CmBGAL1*	ERE^2^, P-box, TCA-element^2^, TGA-element, CGTCA-motif, TGACG-motif	ARE, WUN-motif	Box 4^5^, GA-motif, MRE	AAGAA-motif, CAAT-box^22^, AT-rich element, TATA-box^56^
	*CmBGAL2*	ERE^3^, P-box, TCA-element, CGTCA-motif, GARE-motif, TGACG-motif	MBS^2^, TC-rich repeats, ARE	3-AF1 binding site, TCT-motif^2^	AAGAA-motif, CAAT-box^23^, O2-site, TATA-box^30^
	*CmBGAL3*	ERE, ABRE^2^, P-box, TCA-element	LTR^2^, TC-rich repeats, ARE^2^	G-Box, G-box, Box 4, GT1-motif, GATA-motif, MRE	AAGAA-motif, CAAT-box^43^, O2-site, MBSI, TATA-box^38^
	*CmBGAL4*	ERE^3^, P-box, TCA-element, CGTCA-motif, GARE-motif, TGACG-motif	MBS^2^, TC-rich repeats, ARE	3-AF1 binding site, TCT-motif^2^	AAGAA-motif, CAAT-box^23^, O2-site, TATA-box^30^
B	*CmBGAL18*	ERE^3^	ARE, WUN-motif	Box 4^4^, GT1-motif^2^, chs-CMA1a, ATCT-motif	AAGAA-motif, CAAT-box^38^, MBSI, TATA-box^79^
	*CmBGAL19*	ERE^2^, ABRE^2^, TATC-box, CGTCA-motif^2^, TGACG-motif	LTR^2^, MBS^2^, ARE, WUN-motif	G-Box, G-box, Box 4^7^, TCT-motif^2^, AE-box, ACE	AAGAA-motif^2^, CAAT-box^31^, CCAAT-box, O2-site, TATA-box^35^
	*CmBGAL20*	ERE^2^, ABRE, CGTCA-motif, TGACG-motif, AuxRR-core	ARE, WUN-motif^3^	LAMP-element, GA-motif, ACE, ATC-motif	CAAT-box^36^, AT-rich element, TATA-box^59^
	*CmBGAL21*	ABRE, P-box, TCA-element, TGA-element^2^, CGTCA-motif^2^, GARE-motif, TGACG-motif^2^	TC-rich repeats, ARE^4^, WUN-motif^2^	Box 4^4^, chs-CMA2a, TCT-motif, GATT-motif, ACE	AAGAA-motif, CAAT-box^21^, CAT-box, O2-site^2^, circadian, W box, TATA-box^10^
C	*CmBGAL16*	ERE^4^, ABRE^3^, P-box, TATC-box, TCA-element, GARE-motif	LTR, TC-rich repeats, ARE^3^, WUN-motif^2^	Box II, G-Box^2^, G-box^2^, Box 4, GT1-motif^2^, Gap-box, TCCC-motif, ATCT-motif, GATA-motif, TCT-motif, MRE	AAGAA-motif^3^, CAAT-box^30^, TATA-box^17^
	*CmBGAL17*	ABRE^6^, CGTCA-motif, GARE-motif, TGACG-motif	LTR	G-Box^3^, G-box^5^, Box 4^3^, GT1-motif, TCT-motif, MRE	CAAT-box^20^, O2-site, box S, AT-rich element, TATA-box^38^
D	*CmBGAL13*	ABRE^2^, P-box, CGTCA-motif, TGACG-motif	MBS, TC-rich repeats, ARE^3^	G-box^3^, Box 4, GT1-motif^2^, AT1-motif, GATA-motif^2^, MRE	CAAT-box^31^, O2-site, W box, TATA-box^10^
E	*CmBGAL14*	ERE^4^, ABRE^3^, GARE-motif	LTR, ARE^3^, WUN-motif^2^	G-Box^2^, G-box^2^, Box 4^4^, GT1-motif, chs-CMA1a, TCCC-motif, TCT-motif^2^	CAAT-box^30^, CAT-box, CCAAT-box, GCN4_motif, O2-site, MSA-like, TATA-box^32^
	*CmBGAL15*	ERE^4^, ABRE, TGA-element	MBS, ARE, WUN-motif	G-box, Box 4^2^, 3-AF1 binding site, TCT-motif, GATA-motif	AAGAA-motif, CAAT-box^43^, W box, HD-Zip 1, TATA-box^63^
F	*CmBGAL5*	ERE^3^, ABRE^4^, P-box	MBS, ARE^5^	G-Box^2^, G-box^3^, GT1-motif^2^, I-box^3^, ATCT-motif	CAAT-box^29^, CAT-box^2^, O2-site, box S, TATA-box^24^
	*CmBGAL6*	ERE^4^, TGA-box, TCA-element, TGA-element, CGTCA-motif, TGACG-motif, SARE	ARE^2^, LTR^2^, TC-rich repeats^2^	Box 4^4^, GT1-motif^2^, TCT-motif^2^, ATCT-motif	AAGAA-motif, CAAT-box^35^, O2-site, HD-Zip 1, TATA-box^50^
	*CmBGAL7*	ERE^6^, TGA-element, CGTCA-motif^3^, TGACG-motif^3^	ARE^2^, WUN-motif	GT1-motif, I-box, chs-CMA1a, AAAC-motif	CAAT-box^27^, TATA-box^31^
	*CmBGAL8*	P-box^2^, TCA-element, TGA-element, CGTCA-motif^2^, TGACG-motif^2^	ARE^4^	GA-motif, GT1-motif, MRE	GCN4_motif, AAGAA-motif^2^, CAAT-box^41^, W box, TATA-box^45^
	*CmBGAL9*	–	–	–	–
	*CmBGAL10*	ERE^3^, ABRE^2^	ARE^2^	G-Box, G-box, Box 4^4^, GA-motif, GT1-motif, I-box, chs-CMA1a, 3-AF1 binding site, LAMP-element, TCCC-motif, GATA-motif^2^, TCT-motif, MRE	AAGAA-motif, CAAT-box^26^, W box, TATA-box^45^
	*CmBGAL11*	ERE, ABRE	–	G-box, Box 4^3^, GT1-motif, ACE, MRE	CAAT-box^27^, CCGTCC-box, W box, TATA-box^86^
G	*CmBGAL12*	ERE^4^, ABRE, TCA-element^3^, TGA-element, CGTCA-motif, TGACG-motif	ARE^2^, WUN-motif^2^	G-box, Box 4^2^, GT1-motif, LAMP-element, TCT-motif, GATA-motif^2^	AAGAA-motif^2^, CAAT-box^24^, TATA-box^41^

## Discussion

### Gene functional diversity of *CmBGAL*s

The number of *CmBGAL* members in melon (21) is more than Arabidopsis (17) [23], tomato (17) [29, 30], peach (17) [18], apple (13) [17], Japanese pear (8) [22], strawberry (4) [20, 28] and avocado (4) [24], demonstrating that the *CmBGALs* undergone more whole-genome duplication.

The protein subcellular location prediction of the 21 CmBGALs showed that they were mainly located in extracellular space (cell wall), which same as the subcellular location verified of AtBGAL1–5 and AtBGAL12 in Arabidopsis [32], and of Mdβ-Gal1, Mdβ-Gal2, and Mdβ-Gal5 in apple [17], which confirming that the BGALs involved in cell wall metabolism. Whereas some CmBGALs were also predicted located in the plasma membrane and endomembrane system, as the AtBGAL12 was reported also located in the endoplasmic reticulum [32]. Thus, we deduced that the BGALs may participated in the construction of glycoprotein by releasing the β-D-galactosyl. Interestingly, the CmBGAL11 was predicted located in the nucleus which haven’t be reported before, but the realistic subcellular location of it needs to be verified by experiment.

To assess the physiological functions of *CmBGAL*s, the expression pattern of *CmBGAL*s in various tissues were analyzed by qRT-PCR in two melon cultivars ‘HDB’ and ‘HPM’. The results suggesting that most of the *CmBGALs* existed spatial-specific expression, especially in the organs with vigorous cell wall remodeling, like tendril and stem. Similarly, spatial expression was also observed in seventeen *AtBGALs* in Arabidopsis. *AtBGAL1, AtBGAL2, AtBGAL3* and *AtBGAL5* higher expressed in leaves, roots and flowers, *AtBGAL4* primarily expressed in leaves and roots. *AtBGAL9, AtBGAL10* and *AtBGAL17* expressed in leaves and flowers. *AtBGAL8, AtBGAL11, AtBGAL13* and *AtBGAL16* expressed in flowers, *AtBGAL6* was detected in roots [23]. In addition, the *TBG1–7* in tomato also exhibited tissue-specific expression. The *TBG4* highly expressed in roots, *TBG5* exhibited high abundance in leaves and stems, while *TBG6* only strongly expressed in stems [29]. Meanwhile, different temporal-specific expression of *CmBGAL* members were observed in fruits at different developmental stages. Similar phenomena were also observed in tomato and Japanese pear fruit [22, 29].

### CmBGAL members in Clade F play key roles in melon fruit softening

In this study, the candidate *CmBGALs* relating to fruit softening were screen out by significant differences analysis on expression level among all the *CmBGALs* family members between two softening types of melon cultivars ‘HDB’ and ‘HPM’. Finally, the *CmBGAL3* and *CmBGAL4* in Clade A and *CmBGAL5–11* in Clade F were identified (Fig. 8). Besides, the results were confirmed by correlation analysis between the expression level with fruit hardness (Table 3). But interestingly, we found that the softening-related *BGAL*s reported in other species are mainly distributed in Clade A or Clade E (Fig. 1). In tomato, the *TBG4* (Clade A) silencing line showed a 40% firmer than control of red-ripe fruit, and with lower β-Gal level and higher wall galactosyl content during the early stages of ripening [19]. In Japanese pear, the *PpGAL1* and *PpGAL4* in Clade A specific expressed in the ripe fruit, whose mRNA level coincided with β-Gal activity [22]. In avocado, the *AV-GAL1* (*PaGAL1*) involved in fruit softening is distributed in Clade A [24, 33]. In strawberry, the *Faβgal1* (Clade A) expressed increasingly and up to a maximum in red fruits [28], and the *FaβGal4* (Clade E) silencing lines with fruits that were 30% firmer than control at the ripe stage [20]. In peach, the putative softening-related *PpBGAL2* and *PpBGAL16* were distributed in Clade A and E, respectively [18]. Similarly, the apple *Mdβ-Gal1* and *Mdβ-Gal2* in Clade A and *Mdβ-Gal5* in Clade E which upwardly expressed at the later ripening in fruit, particularly in ‘Fuji’ cultivar with lower firmness and higher β-Gal activity [17]. Whereas in this study, the *CmBGAL5–11* distributed in Clade F exhibited a specific surge in the mature fruit of ‘HPM’ were considered as the key *CmBGAL* members contributing to softening. Moreover, the *CmBGAL7–11* in Clade F showed identical spatiotemporal expression patterns, which had never been found in other species before. So, we deduced that the Clade F is a novel fruit softening-related *BGAL* clade for melon. Meantime, the members in Clade F exhibited fewer introns especially in *CmBGAL7–11,* thus we considered that the member in Clade F were more conserved during evolution. However, the function relationship among the members in it are redundant or accumulative seems need to be further studied. Additionally, we noticed that the Clade F in BGAL phylogenetic tree is divided into two subclusters. One just consists of CmBGAL7–11, which demonstrate that the close homologous relationship among them. In the other subcluster, we found that the CmBGAL6 homologized with SlTBG14, PpBGAL15 and MdBGAL6 (Mdβ-Gal6) (Fig. 1), but the function of these genes hasn’t been identified. Meantime, the spatiotemporal expression patterns of members in Clade E (*CmBGAL14* and *CmBGAL15*) were also analyzed (Fig. S4), although the expression of the two genes increased in the mature fruit of ‘HPM’, no significant difference was observed between the two cultivars. Moreover, we also observed the β-Gal activity changes in ‘HDB’ and ‘HPM’ fruit during development, a correlation analysis was made between it with the expression of the nine softening-related candidate members (Table 3). The results showed that the expressions of *CmBGAL5–11* in Clade F were higher correlated to β-Gal activity than *CmBGAL3* or *CmBGAL4* in Clade A in fruit, but all their correlation coefficients did not reach the significant level, since the β-Gal activity was multiple contributed by CmBGALs isoforms.

### CmBGAL12 is a unique member

By conserved domains and motifs analysis, we found that the CmBGAL12 in Clade G is a special member in CmBGALs family, because the CmBGAL12 only have the Glyco_hydro_35 domain but without the GHD and Gal_Lectin domains, despite the absence of the Gal_Lectin domain also happened in CmBGAL2, CmBGAL3 and CmBGAL4 in Clade A. In addition, the motifs analysis revealed that the CmBGAL12 only contained Motif 1 of the ten motifs, making it as the most remarkable member in the CmBGALs family. The phylogenetic analysis showed that the CmBGAL12 is clustered in Clade G having close homologous relationship with AtBGAL17 in Arabidopsis, SlTBG13 in tomato, PpBGAL17 in peach and Md-β-Gal4 in apple (Fig. 1), and the motif analysis result of CmBGAL12 is same to PpBGAL17 [18]. Meanwhile, this kind of member was also discovered in sweet potato (Ibbgal17) [31], which demonstrating that it is a kind of highly conserved member in plant BGALs families. In addition, the spatiotemporal expression analysis of CmBGAL12 shows that it primarily expressed in stem (Fig. S5).

Furthermore, the protein secondary structure prediction for CmBGALs showed that the CmBGAL12 has the highest percentage of random coil (46.73%) and α-helix (25.07%), and the lowest percentage of extended strand (20.98%) among all the CmBGAL members. Meantime the tertiary structural 3D model of CmBGAL12 is the only one differs from others, which based on the ‘c6eonA’ template, but not the ‘c3w5gB’ template. Additionally, the protein-protein association analysis showed the CmBGAL12 is the only node protein in the network, further reflecting that the CmBGAL12 may has unique protein characteristics from others. Disappointedly, any experimental data about this kind of member was unable to find in plant BGALs families, and the physiological function of this kind of BGAL member need to be further studied.

### *Cis*-acting regulation of *CmBGAL* promoters

*Cis*-acting regulatory elements analysis in promoter sequences provides putative regulation pathways of *CmBGALs*. In the promoters of 21 *CmBGALs,* we found most of them contained ERE which responds to ethylene signal, including the nine softening-related members in Clade A and F (except for *CmBGAL8*). The methyl jasmonate-responsive *cis*-acting regulatory elements CGTCA-motif or TGACG-motif were found in the softening-related members *CmBGAL4* and *CmBGAL6–8.* Meanwhile, through GUS assay suggested that the promoter activity of *Mdβ-Gal2* could be induced by ethylene and methyl jasmonate in apple via the ERE and TGACG motif which act as important recognition sites [17]. The abscisic acid-responsive element ABRE were found in *CmBGAL3, CmBGAL5, CmBGAL11* and *CmBGAL10,* and it has been reported that the expression of *VmβGAL1* and *VmβGAL2* in bilberry (*Vaccinium myrtillus* L.) fruit were significantly induced after postharvest treatment with abscisic acid [34]. Similarly, through suppressing key gene *SlNCED1* in abscisic acid biosynthesis which led to a down-regulation of *SlTBG* [35]. The above studies suggested that ethylene, methyl jasmonate and abscisic acid signal may participate in fruit softening through regulating the transcription of *BGALs*. For stress responsiveness, ARE, the *cis*-acting regulatory element essential for the anaerobic induction was found in most *CmBGAL* promoters, which coincident with the results in *PpBGAL* promoters in peach [18]. Additionally, other *cis*-acting regulatory elements related to stress response like WUN-motif, TC-rich repeats, MBS and LTR were also found. Meantime, numerous light-responsive elements were found in the promoters of all the *CmBGAL* members, as well as in the promoters of peach [18] and sweet potato [31] *BGALs* family members. Thus, we deduced that the *BGALs* may participate in cell wall remodeling in plant photomorphogenesis. However, the specific binding transcription factors for these *cis*-acting regulatory elements involved in *CmBGALs* transcriptional regulation still need to be further studied.

## Conclusions

A total of 21 *BGALs* designated as *CmBGAL1-CmBGAL21* were identified genome-wide in melon, clustered into A-G seven clades*.* Among members, three duplications *CmBGAL1*:*CmBGAL3*, *CmBGAL19*:*CmBGAL21*, and *CmBGAL20*:*CmBGAL21* happened during *CmBGALs* family evolution. Conserved domains analysis revealed that besides the Glyco_hydro_35 domain (PF01301), all the *CmBGAL* members also contained the GHD domain (PF17834) except for CmBGAL12, and the Gal_Lectin (PF02140) domain existed in most CmBGALs at the C-termini. The spatiotemporal expression analysis by qRT-PCR suggesting that the *CmBGAL*s are mainly expressed in tissues with vigorous cell wall remodeling, like tendrils and stems. Importantly, a novel clade of members (Clade F) related to melon fruit softening were discovered. Furthermore, the homologous *CmBGAL7–11* exhibited identical spatiotemporal expression patterns may multiple genes leading to melon fruit softening.

## Methods

### Identification of *BGAL* genes in melon

To obtain the candidate *Cucumis melo* L. *BGAL* genes, melon genome v3.6.1 was downloaded from the Cucurbit Genomics Database (CuGenDB) (http://cucurbitgenomics.org/), hidden Markov model (HMM) research against Glyco_hydro_35 domain [PF01301 in Pfam (http://pfam.xfam.org/)] of BGALs was performed by HMMER3 (http://hmmer.janelia.org/) [29]. Subsequently, all sequences were future examined via Simple Modular Architecture Research Tool (SMART) (http://smart.embl-heidelberg.de/) [36], and multiple sequence alignment were performed using DNAMAN software (Lynnon Corporation, Canada) to identify the final *BGAL* members in melon.

### Phylogenetic analysis

The amino acid sequences of BGALs of *Cucumis melo* were downloaded from CuGenDB (http://cucurbitgenomics.org/), of *Arabidopsis thaliana* from TAIR (http://www.arabidopsis.org/), of *Solanum lycopersicum* and *Prunus persica* from Phytozome v13 (https://phytozome.jgi.doe.gov), of *Malus domestica*, *Pyrus pyrifolia*, *Fragaria ananassa* and *Persea americana* from GenBank (https://www.ncbi.nlm.nih.gov/genbank/), respectively. The gene accession numbers of all the *BGAL* genes are shown in Table S2. All the sequences were aligned using MUSCLE [37] and constructed the phylogenetic tree using Maximum Likelihood (ML) method by Jones-Toylar-Thornton (JTT) model [38], uniform rates, gaps date treatment use all sites, ML heuristic method using Nearest-Neighbor-Interchange (NNI), 3 threads by MEGA X software (Institute of Molecular Evolutionary Genetics, USA) [39].

### Gene information and structure analysis

Information of gene accession number and chromosome location of melon *BGALs* were searched from CuGenDB (http://cucurbitgenomics.org/). Amino acids sequence length, molecular weight (Mw), theoretical isoelectric point (pI) and grand average of hydropathicity index (GRAVY) of BGALs were analyzed by the ExPASy ProtParam (https://web.expasy.org/protparam/) [40]. Subcellular location of BGALs was predicted by BUSCA (https://busca.biocomp.unibo.it/) [41]. Gene sequences with intron and coding sequence (CDS) were downloaded from CuGenDB (http://cucurbitgenomics.org/) to analyze the gene structure using Gene Structure Display Server (GSDS) 2.0 (http://gsds.cbi.pku.edu.cn/index.php) [42].

### Chromosomal location and gene duplication analysis

The chromosomal locations of *CmBGALs* were mapped based on the information in melon genome v3.6.1. For syntenic analysis, the relationships between homologs were verified and visualized by the Advanced Circos tool in TBtools software (South China Agricultural University, China) [43].

### Conserved domains and motifs analysis

Conserved domains and signal peptide were analyzed by NCBI Conserved Domain Database (CDD) (http://www.ncbi.nlm.nih.gov/cdd/) [44] and SMART (http://smart.embl-heidelberg.de/) [36]. Motifs were analyzed and visualized by Multiple Em for Motif Elictition (MEME) v 5.4.1 (http://meme-suite.org/tools/meme), set the find number as 10, and other parameters were default [45].

### Prediction of protein secondary and tertiary structure

The protein secondary structure was predicted by Prabi SOPMA (https://npsa-prabi.ibcp.fr/cgi-bin/npsa_automat.plpage=npsa_sopma.html), the tertiary structure was predicted by Protein Homology/analogY Recognition Engine v 2.0 (Phyre^2^) (http://www.sbg.bio.ic.ac.uk/phyre2/html/page.cgi?id=index) [46].

### Protein-protein association network analysis

The protein association network was analyzed by STRING v 11.5 (https://cn.string-db.org) [47] using the multiple sequences search with the organism chosen as *Cucumis melo*.

### *Cis*-acting regulatory elements analysis in promoters

The 1.5 kb upstream sequences from the start codon of *CmBGALs* were defined as promoter regions obtained from CuGenDB (http://cucurbitgenomics.org/), then using PlantCARE (http://bioinformatics.psb.ugent.be/webtools/plantcare/html/) to identify the *cis*-acting regulatory elements [48].

### Plant materials

Two cultivars of melon (*Cucumis melo* var. *makuwa* Makino) named ‘HDB’ and ‘HPM’ obtained commercially with crisp and mealy texture fruit respectively were taken as materials, the code names were abbreviated from their commercial name ‘Hongdaobian’ (Kaifeng Zhongbo Seedling Research Institute, China) and ‘Hongpimian’ (Hebei Baoding Seedling Company, China), respectively. Seven- or eight-leaf aged seedlings were used for sampling of roots, stems, functional leaves and young leaves tissue, which cultivated in an artificial light climatic incubator (Ledian RLD-1500C-4DW, China) with 12 h light (15,000 Lx) and 12 h dark at a temperature of 25 °C/15 °C, humidity of 60%, set six biological replicates. For flowers, tendrils, and fruits at fruitlet, expanding and mature stage sampling, the plants were grown using substrate bag in a greenhouse at Shenyang Agricultural University, Shenyang, Liaoning Province, P.R. China. Single stem training was adopted, and each plant was set three fruits from the tenth node. Fruits at the same node without disease, insect pests and mechanical injury were chosen, three biological replicates were set at each sampling stage. The sarcocarp from the equatorial part of the fruit was sampled, the samples were frozen with liquid nitrogen and stored at − 80 °C.

### Fruit hardness

The hardness of fruit was detected at 20, 25 and 30 days after anthesis and the mature stage (S1 ~ S4) by a texture analyzer (Brookfield CT3, USA) using the texture profile analysis (TPA) model. The sampling and detection methods were adjusted by Bianchi, et al. (2016) [49]. Column-shaped sarcocarp samples with 1.5 cm diameter and 1 cm height were modified from the equatorial section of fruit, then detected using a TA4/1000 (38.1 mm φ) probe under trigger point load as 10 g; test speed as 2 mm/s; return speed as 2 mm/s; 2 cycles, the recovery time between cycles as 3 s; the target deformation as 3 mm. Three technical replicates for each fruit.

### β-galactosidase activity

β-Gal activity of fruit at fruitlet, expanding and mature stage was determined by a kit (Solarbio BC2580, China). As β-Gal decomposed p-nitrophenyl-β-D-pyranogalactoside to p-nitrophenol, which has the maximum absorption at 400 nm, a microplate reader (TECAN Infinite M200 PRO NanoQuant, Switzerland) was used to measure the absorbance. The production of 1 μmol of p-nitrophenol per gram pulp tissue per hour under 37 °C was defined as one enzyme activity unit. Three technical replicates for each sample.

### RNA isolation and qRT-PCR

Total RNA from various tissues was extracted by an ultrapure RNA kit (CWBIO CW0581M, China). RNA was reverse-transcribed into cDNA using the Primer Script RT reagent kit (TaKaRa PrimeScript™ RT Master Mix, Japan). Specific primers for qRT-PCR of *CmBGALs* were designed by the PrimerQuest Tool (https://sg.idtdna.com/PrimerQuest/Home/Index).

qRT-PCR reactions were performed on a Real-Time PCR Thermal Cycler (Analytic Jena AG qTOWER^3^ G, Germany) using TransStart Top Green qPCR SuperMix (TransGen Biotech, China). PCR program as follows: initial denaturation at 95 °C for 30 s, 40 cycles of 95 °C for 5 s and 60 °C for 34 s, and melt for 15 s. A *Cucumis melo* ribosomal RNA gene (*18S*) was used as an endogenous control for normalization. The gene relative expression was calculated with the 2^−ΔΔCt^ method [50]. Each sample was analyzed in triplicate. All primer sequences are listed in Table S3.

### Statistical analysis

Microsoft Excel 365 was used to process the data. Significant differences between the means were compared by Tukey test using the Paired Comparison Plot App in Origin 2021 software (OriginLab, USA). The correlation analysis was conducted by SPSS Statistics 24 software (IBM, USA). The heatmap and bar chart were drawn by Origin 2021 software. The conserved domains distribution diagram (Fig. 4A) was drawn by Microsoft Power point 365 referred to Chandrasekar and van der Hoorn (2016) [30].

## Supplementary Information

**Additional file 1.**

**Additional file 2.**

**Additional file 3.**

**Additional file 4.**

**Additional file 5.**

**Additional file 6.**

**Additional file 7.**

**Additional file 8.**

**Additional file 9.**

## Data Availability

The datasets analyzed during the current study are available in the CuGenDB, TAIR, Phytozome v13 and GenBank repository, the gene accession numbers of *Cucumis melo* from CuGenDB (http://cucurbitgenomics.org/) are shown in Table 1, The gene accession numbers of *Arabidopsis thaliana* from TAIR (http://www.arabidopsis.org/), of *Solanum lycopersicum* and *Prunus persica* from Phytozome v13 (https://phytozome.jgi.doe.gov), of *Malus domestica*, *Pyrus pyrifolia*, *Fragaria ananassa* and *Persea americana* from GenBank (https://www.ncbi.nlm.nih.gov/genbank/) respectively are shown in Table S2.

## Abbreviations

β-Gal: β-galactosidase
CDS: Coding sequence
CuGenDB: Cucurbit Genomics Database
HMM: Hidden Markov model
GH35: Glycosyl hydrolase 35
GRAVY: Grand average of hydropathicity index
Mw: Molecular weight
PG: Polygalacturonase
pI: Theoretical isoelectric point
PME: Pectin methylesterase
qRT-PCR: Quantitative real-time
RG-I: Rhamnogalacturonan-I
TPA: Texture profile analysis

## References

[1] Vicente AR, Saladié M, Rose JKC, Labavitch JM (2007). The linkage between cell wall metabolism and fruit softening: looking to the future. J Sci Food Agr.

[2] Brummell DA, Harpster MH (2001). Cell wall metabolism in fruit softening and quality and its manipulation in transgenic plants. Plant Mol Biol.

[3] Bartley IM (1974). β-Galactosidase activity in ripening apples. Phytochemistry..

[4] Carrington CMS, Pressey R (1996). β-galactosidase II activity in relation to changes in cell wall galactosyl composition during tomato ripening. J Am Soc Hortic Sci.

[5] Pressey R (1983). β-Galactosidases in ripening tomatoes. Plant Physiol.

[6] Ranwala AP, Suematsu C, Masuda H (1992). The role of β-galactosidases in the modification of cell wall components during muskmelon fruit ripening. Plant Physiol.

[7] De Veau EJI, Gross KC, Huber DJ, Watada AE (1993). Degradation and solubilization of pectin by β-galactosidases purified from avocado mesocarp. Physiol Plantarum.

[8] Ross GS, Redgwell RJ, MacRae EA (1993). Kiwifruit β-galactosidase: Isolation and activity against specific fruit cell-wall polysaccharides. Planta..

[9] Kitagawa Y, Kanayama Y, Yamaki S (1995). Isolation of β-galactosidase fractions from Japanese pear: Activity against native cell wall polysaccharides. Physiol Plantarum..

[10] Lazan H, Ng S-Y, Goh L-Y, Ali ZM (2004). Papaya β-galactosidase/galactanase isoforms in differential cell wall hydrolysis and fruit softening during ripening. Plant Physiol Bioch..

[11] Ng JKT, Schröder R, Brummell DA, Sutherland PW, Hallett IC, Smith BG, Melton LD, Johnston JW (2015). Lower cell wall pectin solubilisation and galactose loss during early fruit development in apple (*Malus x domestica*) cultivar ‘Scifresh’ are associated with slower softening rate. J Plant Physiol.

[12] Dwevedi A, Kayastha AM (2010). Plant β-galactosidases: physiological significance and recent advances in technological applications. J Plant Biochem Biot.

[13] Buckeridge MS, Reid JS (1994). Purification and properties of a novel beta-galactosidase or exo-(1→4)-beta-D-galactanase from the cotyledons of germinated *Lupinus angustifolius* L. seeds. Planta..

[14] Dwevedi A, Kayastha AM (2009). A β-galactosidase from pea seeds (*Ps*BGAL): purification, stabilization, catalytic energetics, conformational heterogeneity, and its significance. J Agr Food Chem.

[15] Martín I, Hernández-Nistal J, Albornos L, Labrador E, Dopico B (2013). βIII-Gal is involved in galactan reduction during phloem element differentiation in chickpea stems. Plant Cell Physiol.

[16] Tanimoto E, Igari M (1976). Correlation, between β-galactosidase and auxin-induced elongation growth in etiolated pea stems. Plant Cell Physiol..

[17] Yang H, Liu J, Dang M, Zhang B, Li H, Meng R (2018). Analysis of β-galactosidase during fruit development and ripening in two different texture types of apple cultivars. Front Plant Sci.

[18] Guo S, Song J, Zhang B, Jiang H, Ma R, Yu M (2018). Genome-wide identification and expression analysis of beta-galactosidase family members during fruit softening of peach [*Prunus persica* (L.) Batsch]. Postharvest Biol Tec..

[19] Smith DL, Abbott JA, Gross KC (2002). Down-regulation of tomato β-galactosidase 4 results in decreased fruit softening. Plant Physiol.

[20] Paniagua C, Blanco-Portales R, Barceló-Muñoz M, García-Gago JA, Waldron KW, Quesada MA (2015). Antisense down-regulation of the strawberry β-galactosidase gene *FaβGal4* increases cell wall galactose levels and reduces fruit softening. J Exp Bot.

[21] Henrissat B (1998). Glycosidase families. Biochem Soc T..

[22] Tateishi A, Nagashima K, Mathooko FM, Mwaniki MW, Kubo Y, Inaba A (2005). Differential expression of members of the β-galactosidase gene family during japanese pear (*Pyrus pyrifolia* L.) fruit growth and on-tree ripening. J Am Soc Hortic Sci.

[23] Ahn YO, Zheng M, Bevan DR, Esen A, Shiu SH, Benson J (2007). Functional genomic analysis of *Arabidopsis thaliana* glycoside hydrolase family 35. Phytochemistry..

[24] Tateishi A, Shiba H, Ogihara J, Isobe K, Nomura K, Watanabe K (2007). Differential expression and ethylene regulation of β-galactosidase genes and isozymes isolated from avocado (*Persea americana* Mill.) fruit. Postharvest Biol Tec.

[25] Tanthanuch W, Chantarangsee M, Maneesan J, Ketudat-Cairns J. Genomic and expression analysis of glycosyl hydrolase family 35 genes from rice (*Oryza sativa* L.). BMC Plant Biol. 2008;8(1):84. 10.1186/1471-2229-8-84 PMC2531105 18664295

[26] Liu J, Gao M, Lv M, Cao J (2013). Structure, evolution, and expression of the β-galactosidase gene family in *brassica campestris* ssp. *chinensis*. Plant Mol Biol Rep.

[27] Hobson N, Deyholos MK (2013). Genomic and expression analysis of the flax (*Linum usitatissimum*) family of glycosyl hydrolase 35 genes. BMC Genomics.

[28] Trainotti L, Spinello R, Piovan A, Spolaore S, Casadoro G (2001). β-Galactosidases with a lectin-like domain are expressed in strawberry. J Exp Bot.

[29] Smith DL, Gross KC (2000). A family of at least seven β-galactosidase genes is expressed during tomato fruit development. Plant Physiol.

[30] Chandrasekar B, van der Hoorn RAL (2016). Beta galactosidases in Arabidopsis and tomato-a mini review. Biochem Soc T.

[31] Hou F, Du T, Qin Z, Xu T, Li A, Dong S (2021). Genome-wide in silico identification and expression analysis of beta-galactosidase family members in sweetpotato [*Ipomoea batatas* (L.) Lam]. BMC Genomics.

[32] Moneo-Sánchez M, Izquierdo L, Martín I, Labrador E, Dopico B (2016). Subcellular location of *Arabidopsis thaliana* subfamily a1 β-galactosidases and developmental regulation of transcript levels of their coding genes. Plant Physiol Bioch.

[33] Tateishi A, Inoue H, Yamaki S (2002). Cloning and expression of β-galactosidase cDNA related to softening of avocado (*Persea americana*) fruit. J Jpn Soc Hortic Sci.

[34] Karppinen K, Tegelberg P, Häggman H, Jaakola L (2018). Abscisic acid regulates anthocyanin biosynthesis and gene expression associated with cell wall modification in ripening bilberry (*Vaccinium myrtillus* L.) fruits. Front. Plant Sci.

[35] Sun L, Sun Y, Zhang M, Wang L, Ren J, Cui M (2012). Suppression of 9-cis-epoxycarotenoid dioxygenase, which encodes a key enzyme in abscisic acid biosynthesis, alters fruit texture in transgenic tomato. Plant Physiol.

[36] Letunic I, Khedkar S, Bork P (2020). SMART: recent updates, new developments and status in 2020. Nucleic Acids Res.

[37] Edgar RC (2004). MUSCLE: multiple sequence alignment with high accuracy and high throughput. Nucleic Acids Res.

[38] Jones DT, Taylor WR, Thornton JM (1992). The rapid generation of mutation data matrices from protein sequences. Bioinformatics..

[39] Kumar S, Stecher G, Li M, Knyaz C, Tamura K (2018). MEGA X: Molecular evolutionary genetics analysis across computing platforms. Mol Biol Evol.

[40] Gasteiger E, Hoogland C, Gattiker A, Se D, Wilkins MR, Appel RD, Walker JM (2005). Protein identification and analysis tools on the ExPASy server. The proteomics protocols handbook.

[41] Savojardo C, Martelli Pier L, Fariselli P, Profiti G, Casadio R (2018). BUSCA: an integrative web server to predict subcellular localization of proteins. Nucleic Acids Res.

[42] Hu B, Jin J, Guo A-Y, Zhang H, Luo J, Gao G (2014). GSDS 2.0: an upgraded gene feature visualization server. Bioinformatics..

[43] Chen C, Chen H, Zhang Y, Thomas HR, Frank MH, He Y (2020). TBtools: an integrative toolkit developed for interactive analyses of big biological data. Mol Plant.

[44] Lu S, Wang J, Chitsaz F, Derbyshire MK, Geer RC, Gonzales NR (2019). CDD/SPARCLE: the conserved domain database in 2020. Nucleic Acids Res.

[45] Bailey TL, Elkan C (1994). Fitting a mixture model by expectation maximization to discover motifs in biopolymers. Proc Int Conf Intell Syst Mol Biol.

[46] Kelley LA, Mezulis S, Yates CM, Wass MN, Sternberg MJE (2015). The Phyre2 web portal for protein modeling, prediction and analysis. Nat Protoc.

[47] Szklarczyk D, Gable AL, Nastou KC, Lyon D, Kirsch R, Pyysalo S (2020). The STRING database in 2021: customizable protein-protein networks, and functional characterization of user-uploaded gene/measurement sets. Nucleic Acids Res.

[48] Lescot M, Déhais P, Thijs G, Marchal K, Moreau Y, Van de Peer Y (2002). PlantCARE, a database of plant *cis*-acting regulatory elements and a portal to tools for in silico analysis of promoter sequences. Nucleic Acids Res.

[49] Bianchi T, Guerrero L, Gratacós-Cubarsí M, Claret A, Argyris J, Garcia-Mas J, Hortós M (2016). Textural properties of different melon (*Cucumis melo* L.) fruit types: Sensory and physical-chemical evaluation. Sci Hortic.

[50] Livak KJ, Schmittgen TD (2001). Analysis of relative gene expression data using real-time quantitative PCR and the 2^-ΔΔCT^ method. Methods..

