# Supplementary material for: Beta-galactosidase gene family genome-wide identification and expression analysis of members related to fruit softening in melon (Cucumis melo L.)
Source: BMC Genomics. 2022 Dec 2;23:795. doi: 10.1186/s12864-022-09006-5 (PMC9716742; doi:10.1186/s12864-022-09006-5)
Supplement: Supplementary file 1 — Additional file 1. [file 12864_2022_9006_MOESM1_ESM.pdf]

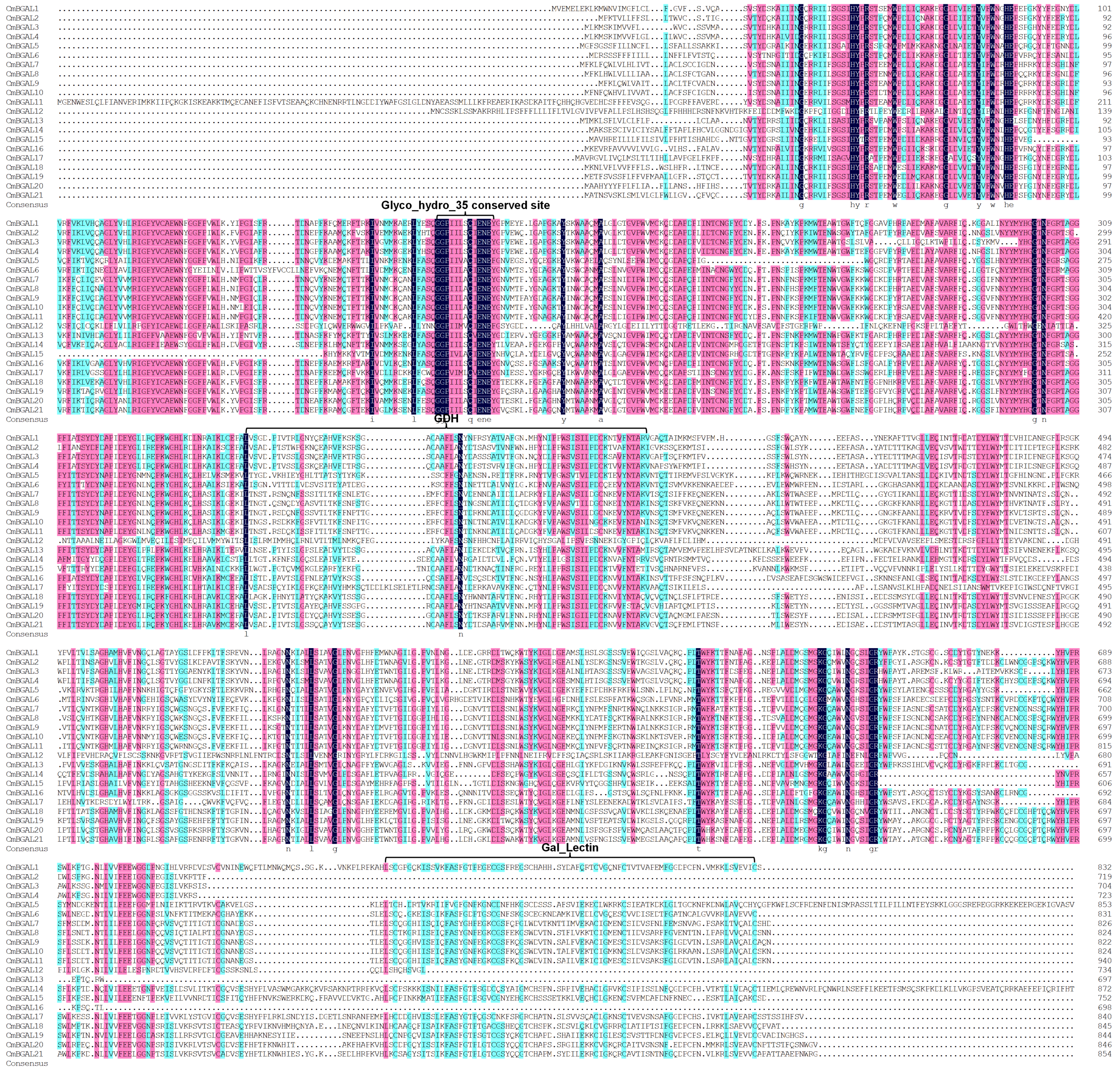

Additional file 1: Figure S1 Multiple sequence alignment of amino acid sequences of CmBGALs. Different shading colors represent homology level (navy blue: 100%; magenta: ≥ 75%; cyan: ≥ 50%)
