# Supplementary material for: Beta-galactosidase gene family genome-wide identification and expression analysis of members related to fruit softening in melon (Cucumis melo L.)
Source: BMC Genomics. 2022 Dec 2;23:795. doi: 10.1186/s12864-022-09006-5 (PMC9716742; doi:10.1186/s12864-022-09006-5)
Supplement: Supplementary file 2 — Additional file 2. [file 12864_2022_9006_MOESM2_ESM.docx]

**Additional file 6: Table S1** Protein conserved domain information in CmBGAL members

| Name | Accession | Description |
| --- | --- | --- |
| Glyco_hydro_35 | pfam01301 | Glycosyl hydrolases family 35 |
| GHD | Pfam17834 | Beta-sandwich domain in beta galactosidase; This entry corresponds to a beta sandwich like domain found in glycosyl hydrolase family 35 beta galactosidase enzymes |
| Gal_Lectin | Pfam02140 | Galactose binding lectin domain |
| CBFD_NFYB_HMF | Pfam00808 | Histone-like transcription factor (CBF/NF-Y) and archaeal histone; This family includes archaebacterial histones and histone like transcription factors from eukaryotes |
