# Supplementary material for: Beta-galactosidase gene family genome-wide identification and expression analysis of members related to fruit softening in melon (Cucumis melo L.)
Source: BMC Genomics. 2022 Dec 2;23:795. doi: 10.1186/s12864-022-09006-5 (PMC9716742; doi:10.1186/s12864-022-09006-5)
Supplement: Supplementary file 3 — Additional file 3. [file 12864_2022_9006_MOESM3_ESM.pdf]

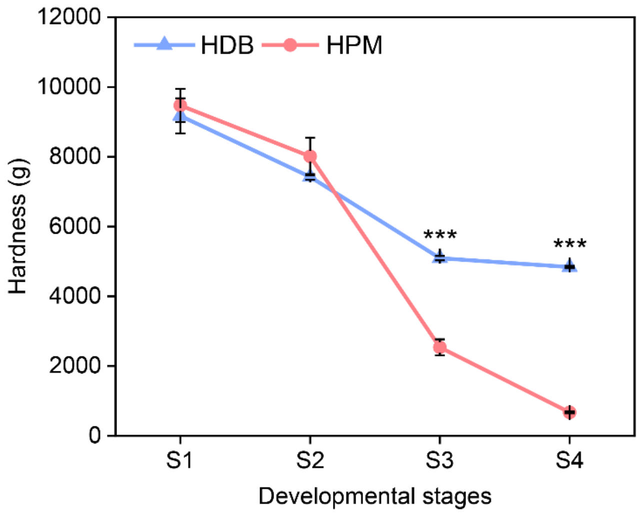

Additional file 2: Figure S2 Hardness change of ‘HDB’ and ‘HPM’ fruit during development. S1~S3 represent 20, 25 and 30 days after anthesis, S4 represents the mature stage. The vertical bars indicate the standard error of the means of triplicates. Significant differences were compared by the Tukey test with * $P < 0.05$, ** $P < 0.01$ and *** $P < 0.001$ between the means of the two cultivars on the same developmental stage
