# Supplementary material for: Beta-galactosidase gene family genome-wide identification and expression analysis of members related to fruit softening in melon (Cucumis melo L.)
Source: BMC Genomics. 2022 Dec 2;23:795. doi: 10.1186/s12864-022-09006-5 (PMC9716742; doi:10.1186/s12864-022-09006-5)
Supplement: Supplementary file 4 — Additional file 4. [file 12864_2022_9006_MOESM4_ESM.pdf]

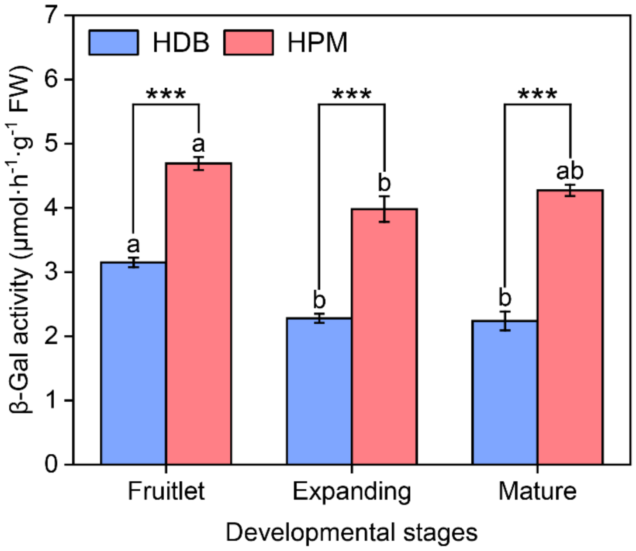

Additional file 3: Figure S3 β-galactosidase activity change of 'HDB' and 'HPM' fruit during development. The vertical bars indicate the standard error of the means of triplicates. Significant differences were compared by Tukey test with * $P < 0.05$, ** $P < 0.01$ and *** $P < 0.001$ between the means of the two cultivars at the same developmental stage. Different letters on each column indicate significant differences between the means among different developmental stages within each cultivar at $P < 0.05$ level
