# Supplementary material for: Beta-galactosidase gene family genome-wide identification and expression analysis of members related to fruit softening in melon (Cucumis melo L.)
Source: BMC Genomics. 2022 Dec 2;23:795. doi: 10.1186/s12864-022-09006-5 (PMC9716742; doi:10.1186/s12864-022-09006-5)
Supplement: Supplementary file 6 — Additional file 6. [file 12864_2022_9006_MOESM6_ESM.pdf]

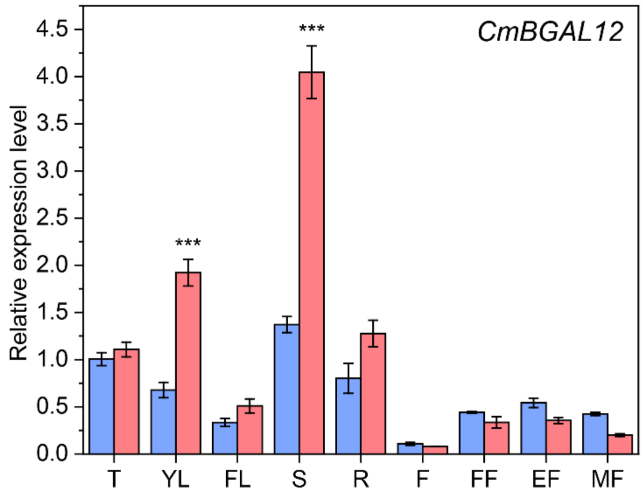

Additional file 5: Figure S5 Relative expression level of *CmBGAL12* of ‘HDB’ and ‘HPM’ in various tissues. T: tendril; YL: young leaf; FL: functional leaf; S: stem R: root; F: flower; FF: fruitlet fruit; EF: expanding fruit; MF: mature fruit. The vertical bars indicate the standard error of the means of triplicates. Significant differences between the means were compared by Tukey test with * $P < 0.05$, ** $P < 0.01$ and *** $P < 0.001$
