# Supplementary material for: Beta-galactosidase gene family genome-wide identification and expression analysis of members related to fruit softening in melon (Cucumis melo L.)
Source: BMC Genomics. 2022 Dec 2;23:795. doi: 10.1186/s12864-022-09006-5 (PMC9716742; doi:10.1186/s12864-022-09006-5)
Supplement: Supplementary file 7 — Additional file 7. [file 12864_2022_9006_MOESM7_ESM.docx]

**Additional file 7: Table S2** Gene accession numbers of identified *BGAL* members in *Arabidopsis thaliana*, *Solanum lycopersicum*, *Prunus persica*, *Malus domestica*, *Pyrus pyrifolia*, *Fragaria ananassa* and *Persea americana*

| Gene | Accession | Gene | Accession | Gene | Accession |
| --- | --- | --- | --- | --- | --- |
| *AtBGAL1* | AT3G13750 | *SlTBG11* | Solyc04g080840 | *MdBGAL4* (*Mdβ-Gal4*) | XM_008353123 |
| *AtBGAL2* | AT3G52840 | *SlTBG12* | Solyc06g062580 | *MdBGAL5* (*Mdβ-Gal5*) | XM_008340441 |
| *AtBGAL3* | AT4G36360 | *SlTBG13* | Solyc06g062660 | *MdBGAL6* (*Mdβ-Gal6*) | XM_008339663 |
| *AtBGAL4* | AT5G56870 | *SlTBG14* | Solyc07g042220 | *MdBGAL7* (*Mdβ-Gal7*) | XM_008395970 |
| *AtBGAL5* | AT1G45130 | *SlTBG15* | Solyc10g055470 | *MdBGAL8* (*Mdβ-Gal8*) | XM_008395969 |
| *AtBGAL6* | AT5G63800 | *SlTBG16* | Solyc11g018490 | *MdBGAL9* (*Mdβ-Gal9*) | XM_008393104 |
| *AtBGAL7* | AT5G20710 | *SlTBG17* | Solyc11g018500 | *MdBGAL10* (*Mdβ-Gal10*) | XM_008385587 |
| *AtBGAL8* | AT2G28470 | *PpBGAL1* | Prupe.3G298200.1 | *MdBGAL11* (*Mdβ-Gal11*) | XM_008379521 |
| *AtBGAL9* | AT2G32810 | *PpBGAL2* | Prupe.3G050200.1 | *MdBGAL12* (*Mdβ-Gal12*) | XM_008378967 |
| *AtBGAL10* | AT5G63810 | *PpBGAL3* | Prupe.7G163100.1 | *MdBGAL13* (*Mdβ-Gal13*) | XM_008360184 |
| *AtBGAL11* | AT4G35010 | *PpBGAL4* | Prupe.1G372800.1 | *PpyGAL1* (*PpGAL1*) | AB046543 |
| *AtBGAL12* | AT4G26140 | *PpBGAL5* | Prupe.8G089500.1 | *PpyGAL2* (*PpGAL2*) | AB190363 |
| *AtBGAL13* | AT2G16730 | *PpBGAL6* | Prupe.4G279500.1 | *PpyGAL3* (*PpGAL3*) | AB190364 |
| *AtBGAL14* | AT4G38590 | *PpBGAL7* | Prupe.4G038600.1 | *PpyGAL4* (*PpGAL4*) | AB190365 |
| *AtBGAL15* | AT1G31740 | *PpBGAL8* | Prupe.6G099700.1 | *PpyGAL5* (*PpGAL5*) | AB190366 |
| *AtBGAL16* | AT1G77410 | *PpBGAL9* | Prupe.1G160600.1 | *PpyGAL6* (*PpGAL6*) | AB190367 |
| *AtBGAL17* | AT1G72990 | *PpBGAL10* | Prupe.4G278500.1 | *PpyGAL7* (*PpGAL7*) | AB190368 |
| *SlTBG1* | Solyc12g044880 | *PpBGAL11* | Prupe.1G009200.1 | *PpyGAL8* (*PpGAL8*) | AB190369 |
| *SlTBG2* | Solyc09g092160 | *PpBGAL12* | Prupe.7G210000.1 | *FaBGAL1* (*Faβgal1*) | AJ278703 |
| *SlTBG3* | Solyc03g121540 | *PpBGAL13* | Prupe.1G009300.1 | *FaBGAL2* (*Faβgal2*) | AJ278704 |
| *SlTBG4* | Solyc12g008840 | *PpBGAL14* | Prupe.8G164800.1 | *FaBGAL3* (*Faβgal3*) | AJ278705 |
| *SlTBG5* | Solyc11g069270 | *PpBGAL15* | Prupe.7G194500.1 | *FaBGAL4* (*FaβGal4*) | KR189030 |
| *SlTBG6* | Solyc02g084720 | *PpBGAL16* | Prupe.1G492800.1 | *PaGAL1* (*AV-GAL1*) | AB061017 |
| *SlTBG7* | Solyc03g019890 | *PpBGAL17* | Prupe.3G048800.1 | *PaGAL2* | AB252827 |
| *SlTBG8* | Solyc01g110000 | *MdBGAL1* (*Mdβ-Gal1*) | L29451 | *PaGAL3* | AB252828 |
| *SlTBG9* | Solyc01g111540 | *MdBGAL2* (*Mdβ-Gal2*) | XM_008389715 | *PaGAL4* | AB252829 |
| *SlTBG10* | Solyc02g078950 | *MdBGAL3* (*Mdβ-Gal3*) | XM_008368138 |  |  |

Note: Gene ID of *Arabidopsis thaliana* (*At*) from TAIR (<http://www.arabidopsis.org/>), of *Solanum lycopersicum* (*Sl*) and *Prunus persica* (*Pp*) from Phytozome v13 (<https://phytozome.jgi.doe.gov>), of *Malus domestica* (*Md*), *Pyrus pyrifolia* (*Ppy*), *Fragaria ananassa* (*Fa*) and *Persea americana* (*Pa*) from GenBank (https://www.ncbi.nlm.nih.gov/genbank/), respectively
