# Supplementary material for: Beta-galactosidase gene family genome-wide identification and expression analysis of members related to fruit softening in melon (Cucumis melo L.)
Source: BMC Genomics. 2022 Dec 2;23:795. doi: 10.1186/s12864-022-09006-5 (PMC9716742; doi:10.1186/s12864-022-09006-5)
Supplement: Supplementary file 8 — Additional file 8. [file 12864_2022_9006_MOESM8_ESM.docx]

**Additional file 8: Table S3** Specific primers for quantitative real-time PCR of *CmBGALs* and actin gene

| Gene | Forward primer (5’-3’) | Reverse primer (5’-3’) |
| --- | --- | --- |
| *CmBGAL1* | GCAAGGTTCATACAGAAGGG | CATAAGAAGTAGCGATGAAGGG |
| *CmBGAL2* | CTGTGGAATGGACGAAAGG | AAGCCAATGGTTCCTTACC |
| *CmBGAL3* | GTGGGTTTGTTCTTCTGTAATG | GGAGTGCTTCTTGGGTAATG |
| *CmBGAL4* | GTATGATGAAGGGGGAGAAGTTAT | GAGCAGCCCATTTGGTGTAA |
| *CmBGAL5* | TTGGAATGCCCATGAACC | CACTCAGCACAGACATAAGG |
| *CmBGAL6* | GACGGAGCCTGTCACCCT | CAGTCCCTTACGCATTTGG |
| *CmBGAL7* | CGTCGCATTATCTTCTCAGG | TCAAGTCCACCATCTTTAGC |
| *CmBGAL8* | GTACAGGAGCGAATGTTACC | CGTGGATAGTGGATTGAACC |
| *CmBGAL9* | TACGATTACAACGCCCCACT | AAAAGCAAAACCTCTCCCCT |
| *CmBGAL10* | TGAACCACGACGACGAAAAT | CTCGGCACACACGTAACCAC |
| *CmBGAL11* | TTGGACATGCAAGGAATGG | TGCCAGCAATGAAAGAAGG |
| *CmBGAL12* | TGGTGACGTTGACAATGC | CGCTGTAGTGCTGTATAACC |
| *CmBGAL13* | CCCCCAAAGCAACAACCT | CGCACTTCTGAACGCACAC |
| *CmBGAL14* | CTGGAGCATTTCTTGAGACC | GATAGGCCAACCTTGTATCC |
| *CmBGAL15* | TCATGTGCAAGCAGAAGG | CAGGCTTATAGGGTTTGTTAGG |
| *CmBGAL16* | TGACTTCGAGGGAAGAAAGG | CATTCCGCACACACATAAGG |
| *CmBGAL17* | GGCCTCTACCTTCATTTACG | ATCTCGCAACCACAAAGG |
| *CmBGAL18* | CAGAAGGAGATGAACCTTTGG | GACATTCTGCCCGTTTATCC |
| *CmBGAL19* | TTGGCAGAAATGGAGTTACC | CTGACCTTGTGAAAGAGATCC |
| *CmBGAL20* | ACTACCCCAGAAGCACCCCT | AAGTTCCACTCGGCACAAAC |
| *CmBGAL21* | GTGATGTGCAAGGAAGAGG | ACATTGCAGGTTTGTAGGG |
| *Cm18S* | AAACGGCTACCACATCCA | CACCAGACTTGCCCTCCA |
